# APE1 and NPM1 protect cancer cells from platinum compounds cytotoxicity and their expression pattern has a prognostic value in TNBC

**DOI:** 10.1186/s13046-019-1294-9

**Published:** 2019-07-15

**Authors:** Matilde Clarissa Malfatti, Lorenzo Gerratana, Emiliano Dalla, Miriam Isola, Giuseppe Damante, Carla Di Loreto, Fabio Puglisi, Gianluca Tell

**Affiliations:** 1 Department of Medicine (DAME), University of Udine, Piazzale M. Kolbe 4, 33100 Udine, Italy; 2 Department of Oncology, ASUI Udine SMM University Hospital Udine, Udine, Italy; 3 Department of Pathology, ASUI Udine SMM University Hospital Udine, Udine, Italy; 4 Department of Medical Oncology, Centro di Riferimento Oncologico (CRO), IRCCS, Aviano, Italy

**Keywords:** TNBC, APE1, NPM1, Platinum salts, APE1-inhibitors, TCGA, Biomarker

## Abstract

**Background:**

Triple negative breast cancer (TNBC) is a breast cancer subgroup characterized by a lack of hormone receptors’ expression and no HER2 overexpression. These molecular features both drastically reduce treatment options and confer poor prognosis. Platinum (Pt)-salts are being investigated as a new therapeutic strategy. The base excision repair (BER) pathway is important for resistance to Pt-based therapies. Overexpression of APE1, a pivotal enzyme of the BER pathway, as well as the expression of NPM1, a functional regulator of APE1, are associated with poor outcome and resistance to Pt-based therapies.

**Methods:**

We evaluated the role of NPM1, APE1 and altered NPM1/APE1 interaction in the response to Pt-salts treatment in different cell lines: APE1 knockout (KO) cells, NPM1 KO cells, cell line models having an altered APE1/NPM1 interaction and HCC70 and HCC1937 TNBC cell lines, having different levels of APE1/NPM1. We evaluated the TNBC cells response to new chemotherapeutic small molecules targeting the endonuclease activity of APE1 or the APE1/NPM1 interaction, in combination with Pt-salts treatments. Expression levels’ correlation between APE1 and NPM1 and their impact on prognosis was analyzed in a cohort of TNBC patients through immunohistochemistry. Bioinformatics analysis, using TCGA datasets, was performed to predict a molecular signature of cancers based on APE1 and NPM1 expression.

**Results:**

APE1 and NPM1, and their interaction as well, protect from the cytotoxicity induced by Pt-salts treatment. HCC1937 cells, having higher levels of APE1/NPM1 proteins, are more resistant to Pt-salts treatment compared to the HCC70 cells. A sensitization effect by APE1 inhibitors to Pt-compounds was observed. The association of NPM1/APE1 with cancer gene signatures highlighted alterations concerning cell-cycle dependent proteins.

**Conclusions:**

APE1 and NPM1 protect cancer cells from Pt-compounds cytotoxicity, suggesting a possible improvement of the activity of Pt-based therapy for TNBC, using the NPM1 and APE1 proteins as secondary therapeutic targets. Based on positive or negative correlation with APE1 and NPM1 gene expression levels, we finally propose several TNBC gene signatures that should deserve further attention for their potential impact on TNBC precision medicine approaches.

**Electronic supplementary material:**

The online version of this article (10.1186/s13046-019-1294-9) contains supplementary material, which is available to authorized users.

## Background

Breast cancer (BC) is a heterogeneous disease comprising several biologically different subtypes characterized by distinctive prognosis and potential therapeutic targets [1, 2]. Although gene expression profiling technologies are capable to finely describe four main intrinsic subtypes, the logistic issues in deploying this methodology in common clinical practice has resulted in the widespread adoption of their immunohistochemistry (IHC)- defined approximation, which is still capable of predicting clinical outcome, pattern of metastatization and benefit from distinct types of therapy [3–7]. Among BC subtypes, triple negative breast cancer (TNBC) represents a challenge because of its heterogeneity and the absence of a well-defined druggable target, restricting clinical decision making to chemotherapy [8–10]. Different approaches have been explored to better dissect the TNBC profile in order to optimize therapeutic choices and exploit new strategies, such as genotoxic agents and interference with different DNA repair pathways [11, 12]. Among the therapeutic options already employed in the clinical practice of several tumors including TNBC, platinum (Pt)-based compounds emerge for their ability to interfere with several cellular processes, including DNA replication and transcription [13–15]. The most common Pt-salts include cisplatin (CDDP), carboplatin (CBDCA) and oxaliplatin [16]. Of note, the activity of the single agent CBDCA was investigated through the randomized phase III trial TNT. After a median follow-up of 11 months, no significant improvements were observed in the total population in terms of overall response rate (ORR) with CBDCA versus docetaxel. However, in patients with a germline BRCA1/2 mutation, ORR was more than two-fold higher when treated with CBDCA, with a longer progression-free survival (PFS) [17]. Regarding the molecular effects of Pt-salts, although several mechanisms of action were hypothesized and are still under investigation, the most accredited is the generation of mono-adducts, intra-strand and inter-strand (ICL) DNA cross links, able to distort the DNA backbone causing the formation of toxic single (SSB) and double (DSB) strand breaks [16]. If not efficiently repaired, DNA damages induced by Pt-salts may cause an extensive blockage of the physiological cellular processes, including DNA replication and transcription, resulting, to an ultimate extent, in cell cycle arrest and apoptosis. The preferable DNA repair pathways acting in repairing bulky lesions induced by Pt-salts are the nucleotide excision repair (NER), the homologous recombination (HR) and the Fanconi anemia (FA) pathways [18, 19]. Just recently, a role of base excision repair (BER) pathway has been also hypothesized [19–21]. Typically, BER is active in repairing non-bulky DNA lesions induced by oxidative, alkylating or methylation stressors [22]. Upon the recognition of the damaged base by specific glycosylases, the apurinic/apyrimidinic endonuclease 1 (APE1, also known as REF-1) cleaves the newly generated abasic site allowing the accomplishment of the DNA repair [23]. The pivotal role of APE1 is demonstrated by its function in cellular viability and embryonic development [24], due to its role in DNA repair activity and other recently characterized non-canonical functions. In fact, APE1 also plays an important role as redox effector on many transcriptional factors, such as NF-κB, HIF-1α, STAT-3, PAX8, AP-1 and p53 [23–27], regulating important genes involved in tumor progression. Moreover, new interesting molecular functions involved in RNA metabolism were recently discovered in our laboratory [23], including processing of damaged RNA [28], miRNAs [29] and abasic and oxidized ribonucleotides embedded in DNA [30]. The different functions of APE1 are finely modulated by expression, localization, posttranslational modifications (PTMs) [31–36] and by its protein-protein interactome, as well. Indeed, APE1 localizes into the nucleus with a peculiar nucleolar accumulation, which depends on active rDNA transcription [37–41], but the precise significance of this subnuclear distribution is currently non-completely clear [42]. Interestingly, a proper shuttling from nucleoli to nucleoplasm is essential for an efficient response to genotoxic damage [43]. Up to now, it is well accepted that APE1 subcellular localization changes are associated with several cellular functions, as well as to cancer onset and progression [28, 31, 41]. Interestingly, one of the major determinants of APE1 accumulation within nucleoli is its interaction with nucleophosmin (NPM1), which is modulated by APE1 acetylation [41, 44]. NPM1 is a phosphoprotein, which canonically acts as a central factor in rRNA gene processing and as a chaperone in ribosome biogenesis involved in cell proliferation [45, 46]. Depending on the cellular context, NPM1 may act both as a proto-oncogene and as a tumor suppressor and its perturbations are often involved in tumorigenesis and cancer progression [47, 48]. The *NPM1* gene is also involved in several chromosomal translocation characterizing several tumors and involving genes such as *ALK*, *RAR* and *MLF1* [49]. In addition, an aberrant overexpression of the NPM1 protein is another causing factor of several tumors including colon and ovarian cancers [48, 50, 51]. Notably, its localization has an impact on tumorigenesis. Indeed, NPM1 prevalently localizes within the nucleoli, but it constantly shuttles between the nucleus and the cytoplasm [45, 46, 52, 53]. We have already demonstrated that NPM1, and its localization, have an impact on BER activity. In fact, NPM1 is an important functional regulator of BER factors, specifically controlling levels and localization of BER proteins, including APE1 [43]. Moreover, in acute myeloid leukemia (AML)- associated mutations, the mutated *NPM1* gene determines the formation of an aberrant NPM1 protein (NPM1c+) which re-localizes in the cytoplasm. This mis-localization hampers canonical functions of NPM1 [54–56] and affects APE1 nuclear BER function in cancer cells, through relocalization of APE1 itself in the cytoplasm [41]. Finally, it has been demonstrated that higher levels of APE1, often detected in several cancers, confer acquired resistance to chemotherapeutic agents [57] and that hyperacetylation of APE1 is associated with the TNBC phenotype [31]. For these reasons, APE1 is an emerging promising therapeutic target for cancer treatment [58]. To this aim, research has been recently focused on the interference of APE1 functions, including the AP-endonuclease function (e.g. Compound #3) and the redox function (e.g. APX3330) [59, 60] (Codrich et al., submitted), and on efficiently disrupting the APE1/NPM1 interaction, such as SB206553, Fiduxosin and Spiclomazine [61]. One of our purposes was testing whether the treatment with BER inhibitors could sensitize cancer cells to genotoxic agents [61]. Although partially investigated, the relationship between BER and Pt-salts needs to be further explored [20, 21, 62–68]. Based on the above mentioned evidences, we deemed fundamental to investigate the cytotoxicity induced by the combined treatment of Pt-compounds and APE1- inhibitors, which may have synergistic therapeutic effects in the treatment of cancers such as TNBC [69, 70].

For this reason, starting from the emerging importance of Pt-salts for the treatment of TNBC patients and, in parallel, from the continuously evolving knowledge on APE1 functions, the purpose of this study was to understand the role of APE1, and of its interactor NPM1, in TNBC cell lines treated with Pt-compounds, including CDDP and CBDCA. Specifically, by using different cancer cell lines and specific NPM1- or APE1- gene knockout cell models, we explored: i) the protective role of APE1 and NPM1 in CDDP cytotoxicity and ii) whether the APE1 and NPM1 proteins were modulated in terms of level and subcellular localization upon Pt-compounds treatment in TNBC cancer cells. Moreover, we investigated whether targeting APE1 endonuclease activity or its interaction with NPM1 may sensitize TNBC cancer cells to Pt-compounds treatment. To corroborate our in vitro data, we also considered APE1 and NPM1 levels in a real-world cohort of patients affected by TNBC and explored their potential prognostic impact for further hypothesis-generation and potential clinical utility. Finally, we analyzed the TCGA-BRCA dataset (*n* = 1105), focusing in particular on TNBC patients (*n* = 180), and identified several gene signatures whose expression levels correlated in a positive or negative way with APE1 and NPM1 expression. Notably, we examined the differences in patients that had an overall survival above/below 5 years, or that underwent disease recurrence after at least 1 year from diagnosis, highlighting the genes and the underlying molecular processes that may be involved in this different outcome.

## Methods

### Cell culture and transfection, chemicals and viability assays

HCC70 and HCC1937 breast cancer cells (from ATCC®, Sigma, Milan, Italy) and mouse embryonic fibroblast (MEF) expressing (MEF NPM1^+/+^) or not NPM1 (MEF NPM1^−/−^) [71], were cultured in RPMI 1640 supplemented with 10% (vol/vol) fetal bovine serum (FBS). HCC70 cells carry a germline mutation (R^248^Q) in the DNA binding domain (DBD) on the P53 protein and present a wild type *BRCA1* gene, whereas HCC1937 cells have an acquired mutation (C^306^T) occurring near the tetramerization domain of P53 (amino acids 324–359) and are homozygous for the *BRCA1* ^5382^insC mutation. HCT116 colon cancer cells (from ATCC®, Sigma, Milan, Italy) were cultured in DMEM High Glucose supplemented with 10% (vol/vol) FBS; OCI-AML2 and 3 were cultured in α-MEM supplemented with 20% (vol/vol) FBS [41]; CH12F3 cell lines were cultured in RPMI 1640 supplemented with 10% (vol/vol) FBS, 50 μM β-mercaptoethanol and 25 mM HEPES [72]. All culturing media were also supplemented with 100 U/ml penicillin, 100 mg/ml streptomycin sulphate and 2 mM l-glutamine (EuroClone, Milan, Italy). Transfection of HCC70 cells with FLAG-NPM1 plasmid was performed as explained in [41]. Compound #3 and APE1/NPM1 inhibitors [61] were dissolved in dimethyl sulfoxide (DMSO) as 10 mM stock, cisplatin was dissolved in dimethylformamide (DMF) as 33.3 mM stock and carboplatin was dissolved in water as 13.45 mM stock (SIGMA, Milan, Italy). For the MTS viability assay, 5.0 × 10^3^ cells per well were seeded in 96-well plates and grown for 24 h. After the described treatment in 80 μl, cell viability was measured by using the MTS assay (CellTiter 96® AQueous One Solution Cell Proliferation Assay – Promega, Milan, Italy) and a multiwell reader. Values were read at 490 nm of absorbance and were standardized to wells containing media alone. Cell viability of both OCI-AML and CH12F3 lymphocytes was measured as described in [72]. Differences in viability were analyzed through the Student’s t-test using the GraphPad Prism software. Results were considered statistically significant when p <  0.05. IC_50_ values of each tested drug, and Bliss analysis in combined treatments as well, were obtained by using the Combenefit 2.021 software.

### Preparation and quantification of cell extracts

For the preparation of whole cell extracts, cells were harvested by trypsinization and centrifuged at 1000 rpm for 5 min at RT. The supernatant was removed and the pellet was washed once with ice-cold phosphate-buffered saline (PBS) and then centrifuged again. Cell pellet was re-suspended in lysis buffer containing 50 mM Tris HCl (pH 7.4), 150 mM NaCl, 1 mM EDTA and 1% (wt/vol) Triton X-100 supplemented with 1 mM protease inhibitor cocktail (Sigma, Milan, Italy), 0.5 mM phenylmethylsulfonyl fluoride (PMSF), and cooled in ice for 10 min. After centrifugation at 13,000 x g for 20 min at 4 °C, the pellet was discarded while the supernatant conserved at − 20 °C. The protein concentration was determined using the Bio-Rad protein assay reagent (Bio-Rad, Hercules, CA), using BSA (SIGMA, Milan, Italy) as normalizer.

### Western blotting analysis

To evaluate the protein levels at basal and treated conditions, 15 μg of whole cell extracts were run in a 12% SDS-PAGE gel. Proteins were then transferred to nitrocellulose membranes (Schleicher & Schuell, Keene, NH). Membranes were saturated by incubation for 1 h at RT with 5% (wt/vol) nonfat dry milk in PBS–0.1% (wt/vol) Tween-20 and then incubated o/n with monoclonal APE1 (NB 100–116 Novus Biologicals, Littleton, CO, USA), a polyclonal NPM1 (ab15440 Abcam, Cambridge, UK), a monoclonal NPM1c+ [41] and a monoclonal FLAG (F1804, Sigma, Milan, Italy) at 4 °C. After three washes with PBS–0.1% (wt/vol) Tween-20, membranes were incubated with a IRDye800 labelled secondary antibodies (diluted to 1:10000) for 1 h at RT. Finally, the membranes were washed three times with PBS–0.1% (wt/vol) Tween-20 and the blots were developed using Odissey® CLx Imager (Li-Cor GmbH, Germany) and quantified using Image Studio™, version 5 (Li-Cor GmbH, Germany). Monoclonal β-tubulin (T 0198 SIGMA, Milan, Italy) and polyclonal β-actin (A 2066 SIGMA, Milan, Italy) proteins were used as normalizers. Protein levels were analyzed through the Student’s t-test using the GraphPad Prism software. Results were considered statistically significant when *p* <  0.05.

### Cohort design and statistical analysis

We retrospectively analyzed a cohort of 111 consecutive TNBC patients, treated between 2000 and 2014 at the Department of Oncology of the University Hospital of Udine, Italy. Cases were eligible independently from stage at diagnosis, but patients with a history of secondary malignancy within the last 3 years were not included in the analysis, except for adequately treated basal cell or squamous cell skin cancer, or carcinoma in situ of the cervix. Individual data and information on primary and advanced disease were collected from electronic health records and treated anonymously according to strict privacy standards. The study was evaluated and approved by the Regional Ethics Committee; protocol number 27835. Correlations between cellular levels of cytoplasmic APE1, nuclear APE1 and NPM1 were investigated through the Spearman’s test after normality evaluation with the Shapiro–Wilk test. Associations with clinico-pathological features were explored by the Mann-Whitney U or Kruskal-Wallis tests, as statistically appropriate. For categorical variables, the chi-square test was performed. Overall survival (OS) was defined as the time between breast cancer diagnosis and death for any cause. Event free interval (EFI) was defined as the time between breast cancer diagnosis and local or distant relapse. The prognostic impact of the variables taken into consideration was investigated in the subset of patients that were not diagnosed with de novo metastatic disease. The cumulative incidence method was used to estimate EFI accounting for the presence of death as competing risks. Based on the method of Fine and Gray (1999), univariate competing-risk regression was used to explore which factors were associated with EFI. This model is based on the hazard of the subdistribution and provides a simple relationship between covariates and cumulative incidence [73]. OS was described according to the Kaplan-Meier approach. Univariate Cox regression was used to estimate which factors were associated with OS. Statistical analysis was conducted using the StataCorp 2016 Stata Statistical Software: Release 14.2 (College Station, Texas, USA).

### Immunohistochemistry analysis

Formalin-fixed and paraffin embedded breast cancer tissues from core biopsies or nodulectomies were immunohistochemically stained using the peroxidase/DAB Plus Dako REAL TM EnVision TM Detection System (Dako A/S, Glostrup, Denmark). Heat induced retrieval of antigen epitopes was carried out in a water bath at 98 °C with 0.01 M sodium citrate buffer (pH 6.0) for 30 min. All primary antibodies were incubated for 60 min at RT. The following mouse monoclonal primary antibodies were used: APE1 (13B8E5C2 – Novus Biological 1/200 diluted) and NPM1 (FC-61991 – ThermoFisher Scientific 1:50 diluted). Positive and negative controls for each marker were included in each staining run according to the supplier’s data sheet. Immunostaining was semiquantitatively evaluated by using light microscopy and by scanning the entire section at high-power magnification (× 400). Staining localization (nuclear and cytoplasmic) was scored in parallel leading to the determination of the percentage of nuclear positive cells and the percentage of cytoplasmic positive cells. For NPM1, nucleolar localization was also recorded.

### The cancer genome atlas gene expression analysis

We queried the cBioPortal for Cancer Genomics [74, 75] using the cgdsr package (Jacobsen 2018) [76] inside the R/Bioconductor environment (R Core Team 2017) [77]. Starting from all the available cancer studies (*n* = 169, Version 1.11.3), we focused on those that had RNA-seq data available (*n* = 36). We recovered the relative expression of APE1 and NPM1, expressed in terms of Z-score, for all the patients profiled in every examined cancer study. Afterwards, we used the stats package to calculate the Pearson correlation coefficient between paired samples and the associated *p*-values. Finally, we used the graphics package to draw a barplot summarizing the trend of statistically significant (*p* ≤ 0.05) correlations. Clinical data for TNBC patients from the TCGA-BRCA dataset was obtained using the FirebrowseR R package [78]. Patients with an overall survival above/below five years were identified, as well as those that did or did not undergo disease recurrence after at least one year from diagnosis. For every group of patients, genes having positive or negative correlation (ρs ≥ + 0.5 or ρs ≤ − 0.5, p ≤ 0.05) with both APE1 and NPM1 gene expression levels were selected and functionally characterized.

### Functional analysis

Gene signatures having positive or negative correlation with APE1/NPM1 were functionally characterized using the Cytoscape plugin ClueGO [79, 80] querying the following functional and metabolic databases: CLINVAR_Human-diseases, WikiPathways, KEGG, CORUM-FunCat-MIPS, REACTOME (“Reactions” and “Pathways”) and Gene Ontology (“Biological Process” and “Immune System Process”, Min GO Level = 4 and Max GO Level = 8). A right-sided hypergeometric test (corrected using the Benjamini-Hochberg method to control the false discovery rate) was applied to find enriched terms (adjusted *p* ≤ 0.05). Related terms sharing similar associated genes were fused to reduce redundancy.

## Results

### APE1 and NPM1 proteins protect from CDDP-induced cytotoxicity in different cell line models

**Fig. 1:**
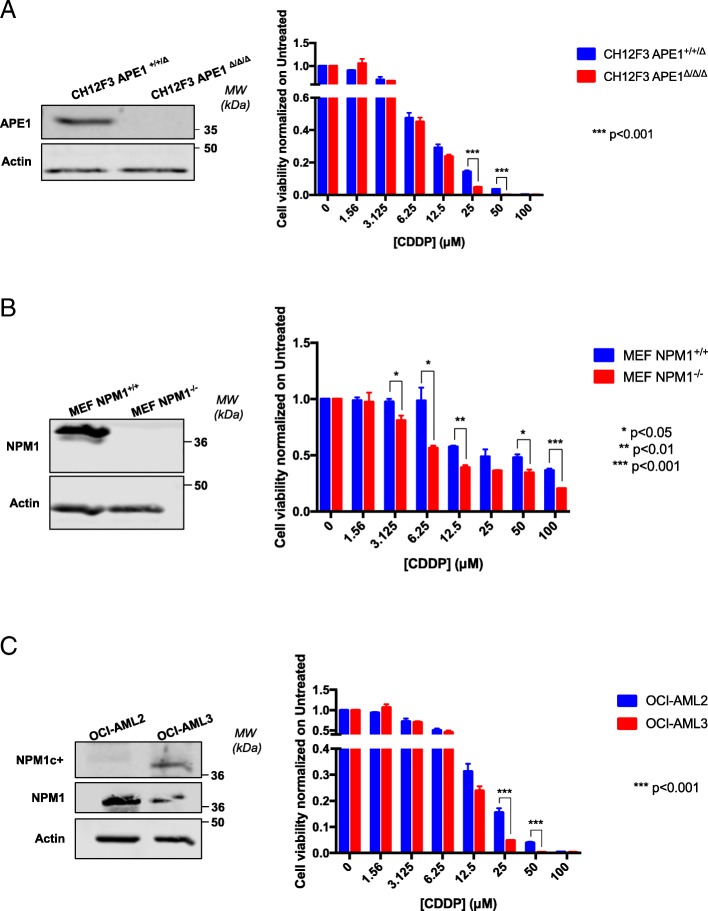
APE1 and NPM1 are involved in the response to CDDP cytotoxicity. **a** Representative western blotting on whole cell extracts (WCE) of CH12F3 cells shows the different amount of APE1 in CH12F3 APE1^+/+/Δ^ and the isogenic knock out CH12F3 APE1^Δ/Δ/Δ^. On the right side of each panel, the Molecular Weights (*MW*), expressed in *kDa*, are indicated. On the left side of each panel, specific antibodies used in the immunoblotting are indicated. Actin was used to normalize APE1 levels. Histograms show the decreased viability of both cell lines treated with the indicated doses (μM) of CDDP for 24 h. Values express the mean viability ± SD from at least three independent replicates. Each value is normalized to the untreated condition. ***p < 0.001. **b** Representative western blotting on whole cell extracts (WCE) of MEF cells shows the different amount of NPM1 in MEF NPM1^+/+^ and the isogenic knock out MEF NPM1^−/−^. On the right side of each panel, the Molecular Weights (*MW*), expressed in *kDa*, are indicated. On the left side of each panel, specific antibodies used in the immunoblotting are indicated. Actin was used to normalize NPM1 levels. Histograms show the decreased viability of both cell lines treated with the indicated doses (μM) of CDDP for 24 h. Values express the mean viability ± SD from at least three independent replicates. Each value is normalized to the untreated condition. *p < 0.05, **p < 0.01, ***p < 0.001. **c** Representative western blotting on whole cell extracts (WCE) of OCI-AML cells shows the presence of NPM1c + in OCI-AML3 compared to the counterpart OCI-AML2. On the right side of each panel, the Molecular Weights (*MW*), expressed in *kDa*, are indicated. On the left side of each panel, specific antibodies used in the immunoblotting are indicated. Actin was used to normalize APE1 levels. Histograms show the decreased viability of both cell lines treated with the indicated doses (μM) of CDDP for 24 h. Values express the mean viability ± SD from at least three independent replicates. Each value is normalized to the untreated condition. ***p < 0.001

We first checked the contribution of the APE1 and NPM1 proteins towards CDDP-induced cell cytotoxicity by using APE1- and NPM1-knockout (KO) cell lines. Specifically, we took advantage of CH12F3 cells expressing APE1 (CH12F3 APE1^+/+/Δ^) and the isogenic KO (CH12F3 APE1^Δ/Δ/Δ^) [72, 81]. Dose response experiments with CDDP (Fig. 1a) clearly demonstrated that CH12F3 APE1^Δ/Δ/Δ^ cells are significantly more sensitive to the treatment than the isogenic control cells expressing APE1. Similarly, in order to test the effect of NPM1 on CDDP-induced cytotoxicity, we used the MEF cells expressing (NPM1^+/+^) or not (NPM1^−/−^) NPM1 [41, 53, 71]. Again, the expression of NPM1 prevents CDDP-induced cytotoxicity, thus exerting a protective function (Fig. 1b). Finally, in order to evaluate the role of a functional interaction of APE1/NPM1 on CDDP-induced cytotoxicity, we used the OCI-AML3 cells characterized by the expression of a haploinsufficient form of the NPM1 protein, called NPM1c+, which aberrantly localized into the cytoplasm. NPM1c + is also associated with a functional impairment of APE1, as a consequence of APE1 aberrant subcellular distribution [41]. By treating OCI-AML3 cells with CDDP, and by comparing their viability with the wild type counterpart OCI-AML2, we clearly observed a significant higher sensitivity of the NPM1c + expressing cell line with respect to the normal isogenic control. All these data demonstrate that, not only the presence of APE1 and NPM1, but also their functional interaction, plays a major role in protecting tumor cells from CDDP-induced cytotoxicity (Fig. 1c).

### Differential sensitivity of TNBC cells to platinum salts depends on NPM1 expression and involves cell-type specific effects on the APE1/NPM1 proteins

**Fig. 2:**
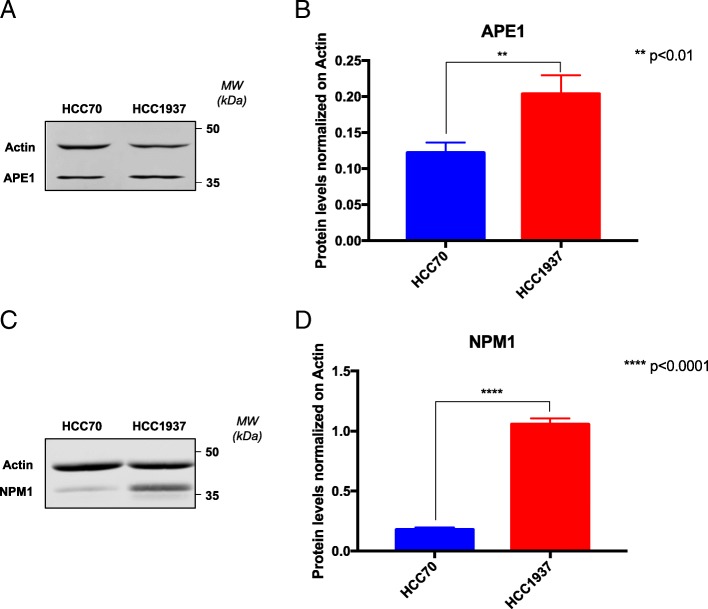
HCC1937 cells show increased levels of APE1 and NPM1 proteins compared to HCC70 cells. Representative western blotting on whole cell extracts of TNBC cells shows the amount of APE1 (**a, b**) and NPM1 (**c, d**) proteins. On the right side of each panel, the Molecular Weights (*MW*), expressed in *kDa*, are indicated. On the left side of each panel, specific antibodies used in the immunoblotting are indicated. Relative graphs (**b, d**) report the difference of each protein, normalized on Actin, between the HCC70 (blue bar) and HCC1937 (red bar) cell lines. **p < 0.01, ****p < 0.0001

**Fig. 3:**
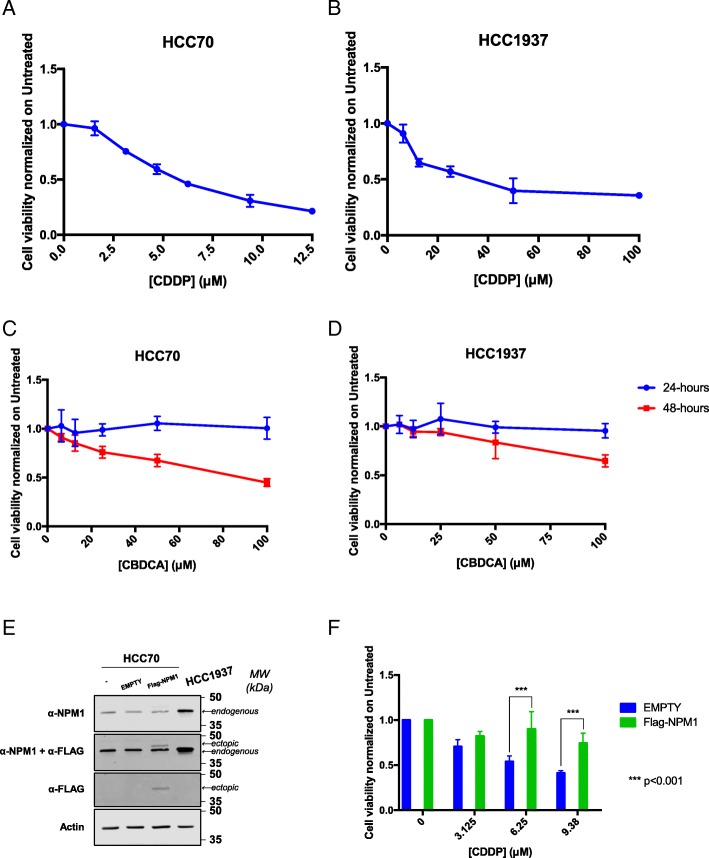
Characterization of the different cytotoxic effect of CDDP and CBDCA on TNBC cells. Dispersion graphs show the decreased viability of HCC70 (**a**) and HCC1937 (**b**) cells when treated with the indicated doses (μM) of CDDP for 24 h. Graphs in (**c**) and (**d**) report the diminished viability of HCC70 and HCC1937 cells, respectively, when treated with the indicated doses (μM) of CBDCA for 24 (blue line) and 48 (red line) hours. **e** Western blotting on whole cell extracts of HCC70 cells transfected with the Flag-NPM1 plasmid and relative control (EMPTY) compared to HCC1937 cells shows the amount of NPM1. On the right side of each panel, the Molecular Weights (*MW*), expressed in *kDa*, are indicated. On the left side of each panel, specific antibodies used in the immunoblotting are indicated. **f** Histogram reporting the viability of HCC70 cells overexpressing NPM1 upon CDDP treatment. Values express the mean viability ± SD from at least three independent replicates. Each value is normalized to the untreated condition. ***p < 0.001

**Fig. 4:**
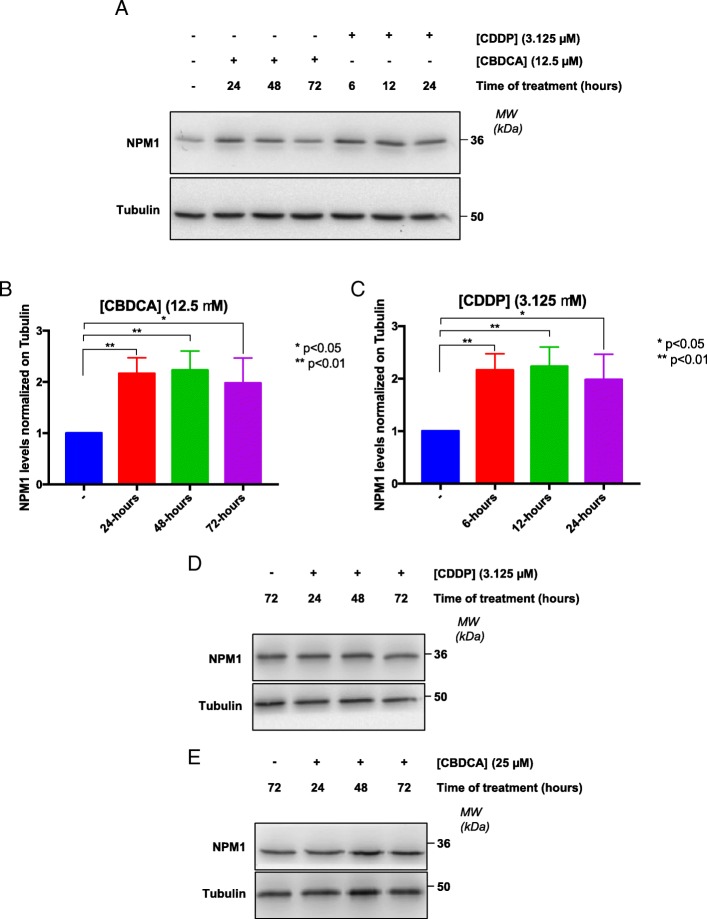
Chronic treatment with Pt-compounds induces an increase of NPM1 in HCC70 cells. **a** Representative western blotting shows the NPM1 protein levels trend in HCC70 cells, chronically treated with CDDP and CBDCA at the indicated concentration for different time-points, as specified on the top of the panel. On the right side of each panel, the Molecular Weights (*MW*), expressed in *kDa*, are indicated. On the left side of each panel, specific antibodies used in the immunoblotting are indicated. **b, c** Histograms reporting the quantitative values corresponding to NPM1 protein amounts upon different time points of CBDCA (**b**) and CDDP (**c**) treatment compared to the basal untreated conditions and normalized on β-tubulin. Values express the mean viability ± SD from at least three independent replicates. *p < 0.05, **p < 0.01. **d, e** Representative western blotting shows the NPM1 protein levels trend in HCC1937 cells, chronically treated with CDDP (**d**) and CBDCA (**e**) at the indicated concentration for different time-points, as specified on the top of the panel. On the right side of each panel, the Molecular Weights (*MW*), expressed in *kDa*, are indicated. On the left side of each panel, specific antibodies used in the immunoblotting are indicated

Two different TNBC cell lines (HCC70 and HCC1937) were chosen as a model for this study, based on their different mutational status of the *TP53* and *BRCA1* genes, two important players in the response to Pt-salts [82, 83]. First, basal levels of APE1 and NPM1 proteins were analyzed in both cell lines. Western blotting analysis revealed that HCC1937 cells were characterized by little though significantly higher (less than two folds) protein levels of APE1 (Fig. 2a) and significantly higher (more than five folds) levels of NPM1 (Fig. 2b) than HCC70 cells. Based on the difference of APE1 and NPM1 protein levels, we evaluated the effect of Pt-compounds on cell survival. We performed a survival assay, upon treatment with CDDP or CBDCA for different time points (Fig. 3 and Table 1). Specifically, as shown in Fig. 3a, b, both cancer cell lines were sensitive to CDDP after 24 h of treatment. However, their response was markedly different and was in agreement with the expression levels of the APE1 and NPM1 proteins; indeed, HCC1937 cells resulted more resistant to CDDP (range 0–100 μM) (Fig. 3b) than HCC70 cells, which were highly sensitive in the 0–12.5 μM range of treatment (Fig. 3a). In the case of CBDCA-treatment, we did not observe any major cytotoxicity after 24 h of treatment (Fig. 3c, d). However, both cell lines showed a significant decrease in survival upon 48h of treatment (red lines in Fig. 3 c, d). As previously observed with CDDP, HCC70 cells were particularly sensitive to the treatment, showing a mortality that reached 60% (SD ± 0.041) at a dose of 100 μM. On the other hand, HCC1937 cells were consistently less sensitive to CBDCA, showing only 40% (SD ± 0.062) mortality at the same dose of treatment. Taken together, these data suggest that the two analyzed TNBC cell lines are differentially responsive to Pt-salts, coherently with the different expression levels of both APE1 and NPM1 proteins. In particular, HCC70 cells resulted much more sensitive to Pt-compounds than HCC1937 cells. For this reason, we tested the protective activity of NPM1 in HCC70 cells by overexpressing NPM1 by using a cell transfection approach, with a plasmid encoding for the wild type flagged form of NPM1 (Fig. 3e-f) [41]. The obtained data clearly demonstrated that the NPM1 overexpression in HCC70 cells (Fig. 3e) significantly decreased their sensitization to CDDP treatment, in agreement with the data obtained with the KO cell models and thus confirming the protective function of NPM1 towards CDDP treatment (Fig. 3f). Moreover, since the nucleolar presence and shuttling of the APE1 and NPM1 proteins might be used as markers of nucleolar stress and of cellular response to CDDP, as previously reported [43], we investigated the phenomenon in both TNBC cell lines. As expected, APE1 and NPM1 proteins re-localized from nucleoli into the nucleoplasm compartment upon Pt-salts treatment. Remarkably, the kinetics of shuttling was different among the two cell lines, with a more prolonged time of nucleolar APE1 emptying in HCC1937 cells compared to HCC70 cells, probably due to their different response to CDDP and CBDCA treatments (Additional file 2: Figure S1 and Additional file 3: Figure S2). In order to further investigate the behavior of NPM1 and APE1 localization upon treatment with Pt-salts, we also analyzed the effect on the total amount of APE1 and NPM1 proteins. After a low dose treatment with both Pt-compounds, HCC70 cells showed an increase of NPM1 expression up to two-fold of the basal levels, independently from the duration of the treatment (Fig. 4a-c and Additional file 4: Figure S3). On the contrary, no changes in APE1 expression were apparent upon CDDP and CBDCA treatments (data not shown). Notably, HCC1937 cells did not show any significant alterations either in NPM1 or APE1 (Fig. 4d-e and not shown). Therefore, the NPM1 protein resulted to be up-regulated by treatment with Pt-compounds in HCC70 cells, only. On the other hand, APE1 protein levels were apparently unaffected by CDDP and CBDCA treatments in both TNBC cell lines. Altogether, these data clearly suggest that the differential sensitivity of TNBC cell lines to Pt-salts depends on NPM1 expression and involves cell-type specific effects on APE1/NPM1 proteins.

**Table 1:** List of IC_50_ values obtained for each TNBC cell type, type of drug, and timing response

Cell line	Drug	Time (hr)	IC_50_ (μM)
HCC70	CDDP	24	5.72
HCC1937			29.6
HCC70	CBDCA	24	*n.d.* (> 100)
		48	85.4
HCC1937		24	*n.d.* (> 100)
		48	*n.d.* (interp = 171)
HCC70	Compound #3	24	2.96
		48	3.17
HCC1937		24	*n.d.* (interp = 6)
		48	3.09
HCC70	Fiduxosin	24	*n.d.* (> 20)
		48	*n.d.* (> 20)
		72	*n.d.* (> 20)
	SB206553	24	*n.d.* (interp = 159)
		48	*n.d.* (interp = 116)
		72	68.1
	Spiclomazine	24	50.2
		48	*n.d.*
		72	*n.d.*
HCC1937	Fiduxosin	24	*n.d.* (> 20)
		48	*n.d.* (> 20)
		72	*n.d.* (> 20)
	SB206553	24	93.1
		48	*n.d.* (> 100)
		72	43.8
	Spiclomazine	24	52.4
		48	48.7
		72	*n.d.*

The analysis carried on by using the Combenefit 2.021 software allowed to calculate the IC_50_ values for each drug tested on the two analyzed TNBC and for different time points. *n.d.* means that the goodness of fit value is too low or a standard Hill equation does not correctly account for the specific agent used. In some cases, the expected interpolated IC_50_ value has been reported

### APE1-inhibition sensitizes TNBC cancer cells to Pt-salts treatment

**Fig. 5:**
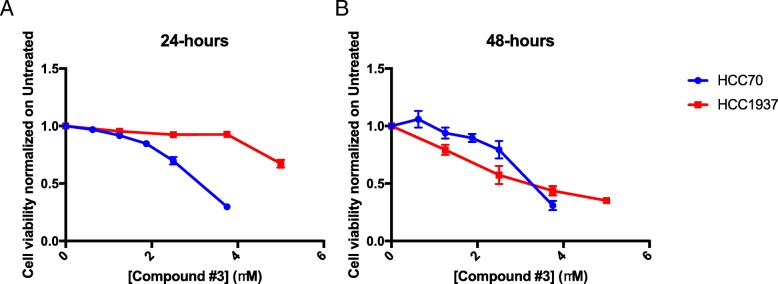
APE1 - inhibitor Compound #3 is differentially toxic in TNBC cell lines. Dispersion graphs show the decreased viability of HCC70 (blue line) and HCC1937 (red line) cells when chronically treated with the indicated doses (μM) of Compound #3 for 24 h (**a**) or 48 h (**b**). Values express the mean viability ± SD from at least three independent replicates. Each value is normalized to the untreated condition

**Fig. 6:**
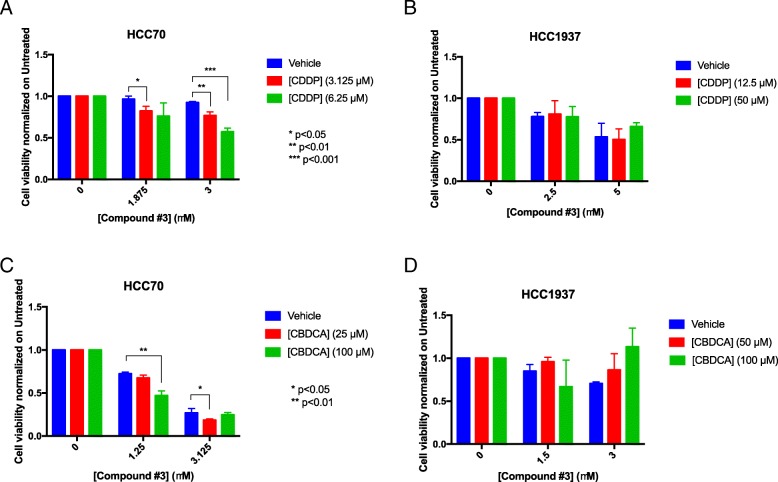
Compound #3 sensitizes only HCC70 cells to Pt-compounds treatment. Histograms show the effect of co-treatments with CDDP (**a, b**) or CBDCA (**c, d**) and Compound #3 on viability of HCC70 (**a-c**) and HCC1937 (**b, d**) cells. Values express the mean viability ± SD from at least three independent replicates. Each value is normalized to the untreated condition. *p < 0.05, **p < 0.01, ***p < 0.001. A Bliss analysis, performed on these data, confirmed the existence of a synergistic effect of Compound #3 and Pt-salts on HCC70 cell line (data not shown)

**Fig. 7:**
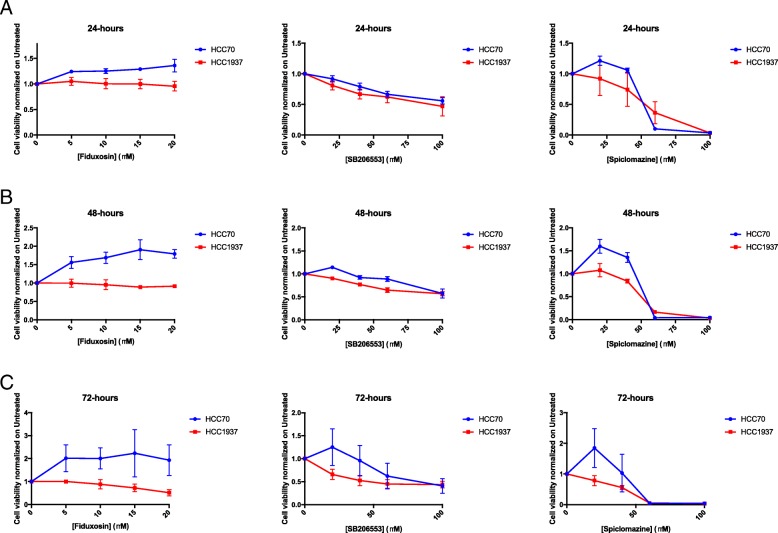
APE1/NPM1 interaction inhibitors are differentially toxic in TNBC cell lines. Dispersion graphs show the decreased viability of HCC70 (blue line) and HCC1937 (red line) cells when chronically treated with the indicated doses (μM) of APE1/NPM1 interaction inhibitors for 24 h (**a**), 48 h (**b**) and 72 h (**c**). Values express the mean viability ± SD from at least three independent replicates. Each value is normalized to the untreated condition

**Fig. 8:**
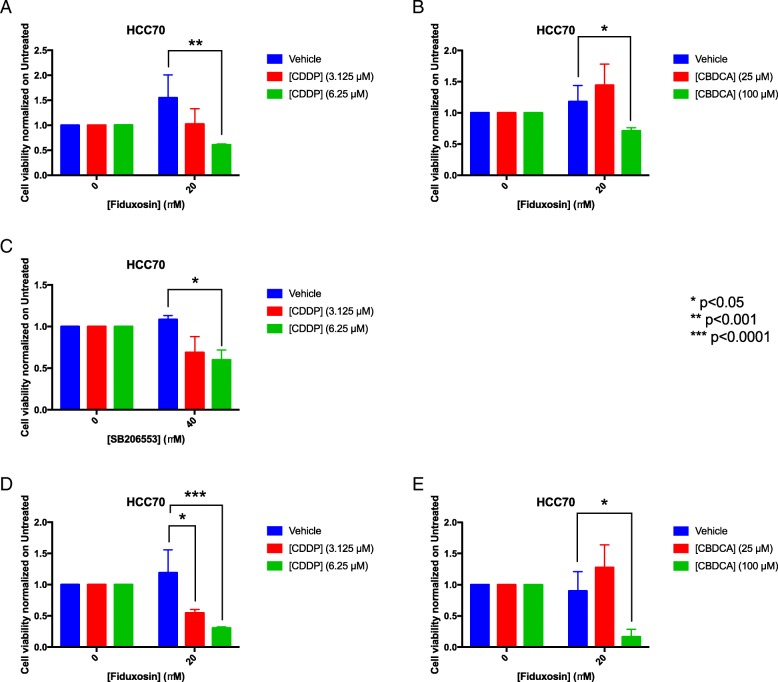
The Fiduxosin and SB206553 APE1/NPM1 interaction-inhibitors sensitize only HCC70 cells to platinum compounds treatment. Histograms show the effect of co-treatments with CDDP (**a, c, d**) or CBDCA (**b**-**e**, **f**) and APE1/NPM1 inhibitors including Fiduxosin (**a**, **b**, **d**, **e**) and SB206553 (**c**), on HCC70 cells viability. Analysis was carried on for 48 h of treatment (**a**,**b**,**c**) or 72 h of treatment (**d**, **e**). Values express the mean viability ± SD from at least three independent replicates. Each value is normalized to the untreated condition. *p < 0.05, **p < 0.001, ***p < 0.0001. A Bliss analysis, performed on these data, confirmed the existence of an additive effect of the APE1/NPM1 inhibitors on HCC70 cell line (data not shown)

In order to address whether APE1 also plays a role in CDDP-cytotoxicity of TNBC cells, we used a targeting strategy with specific APE1 inhibitors. To this purpose, we used several inhibitors of APE1; specifically, we tested Compound #3, highly specific for inhibiting the AP-endonuclease activity of APE1 [59], and three novel drugs, Spiclomazine, Fiduxosin and SB206553, known to interfere with the APE1/NPM1 interaction [61]. The effect of Compound #3 on cell viability was initially assessed, as a single agent, during both 24 h- and 48 h- of treatment (Fig. 5 and Table 1). HCC70 cells resulted more sensitive than HCC1937 cells to CDDP treatment (Fig. 5a), in agreement with a lower expression of APE1 in the former cell line. Although the observed IC_50_ was similar after 48 h for both cell lines (Fig. 5b and Table 1), the cytotoxicity of Compound #3 was higher in HCC70 cells already upon 24 h of treatment (Fig. 5a). Then, we tested the combinatory effect of Compound #3 and Pt-salts treatments (Fig. 6). The experimental setting consisted in a chronic co-treatment with CDDP and Compound #3 for 24 h (Fig. 6a, b) and with CBDCA and Compound #3 for 48 h (Fig. 6c and d). As summarized in the histograms, HCC70 cells showed a significant sensitization effect of Compound #3 on both CDDP (Fig. 6a) and CBDCA (Fig. 6c) treatments. A Bliss analysis, performed on these data, confirmed the existence of a synergistic effect of the two drugs on this cell line (data not shown). On the contrary, such effect was not observed with HCC1937 cells (Fig. 6b and d). Altogether, these data demonstrate that the cytotoxic effect observed by inhibiting the APE1-endonuclease activity is dependent on the APE1 levels expressed by the two TNBC cell lines tested, resulting more cytotoxic for HCC70 than for HCC1937 cell lines. In agreement, the inhibition of APE1-endonuclease activity sensitizes TNBC cell lines to Pt-compounds treatment in an APE1-dependent manner. Afterwards, the same experimental setting was adopted when using APE1/NPM1 inhibitors, either as single agents or in combination with both Pt-compounds. As shown in Fig. 7, the effect of each drug, as single agent, was different between the two TNBC cell lines upon 24 h (Fig. 7a and Table 1), 48 h (Fig. 7b and Table 1) and 72 h (Fig. 7c and Table 1). Initially, we observed a high mortality in both cell lines when treated with Spiclomazine, whereas less mortality was observed when cells were treated with SB206553 or Fiduxosin. We then performed viability assays on combined treatments with Pt-compounds. A Bliss analysis, performed on these data, confirmed the existence of an additive effect of the APE1/NPM1 inhibitors on HCC70 cell line (data not shown), in agreement with the lower APE1/NPM1 levels. Specifically, a high sensitization effect of Fiduxosin with CDDP and CBDCA at 48 and 72 h was observed (Fig. 8a, b and d-e, respectively). Moreover, an effect was also observed when a co-treatment with SB206553 and CDDP was performed (Fig. 8c). Other combinations between APE1/NPM1 inhibitors and Pt-compounds did not sensitize HCC70 cells (Additional file 5: Figure S4). In conclusion, APE1/NPM1 inhibitors resulted to be cytotoxic ‘per se’ independently from the TNBC background. Interestingly, when used in combination with Pt-salts, APE1/NPM1 inhibitors can enhance the effect of crosslinking compounds in a specific TNBC background-dependent manner.

### Characterization of APE1 and NPM1 in a real-world clinical setting is consistent with preclinical models

**Fig. 9:**
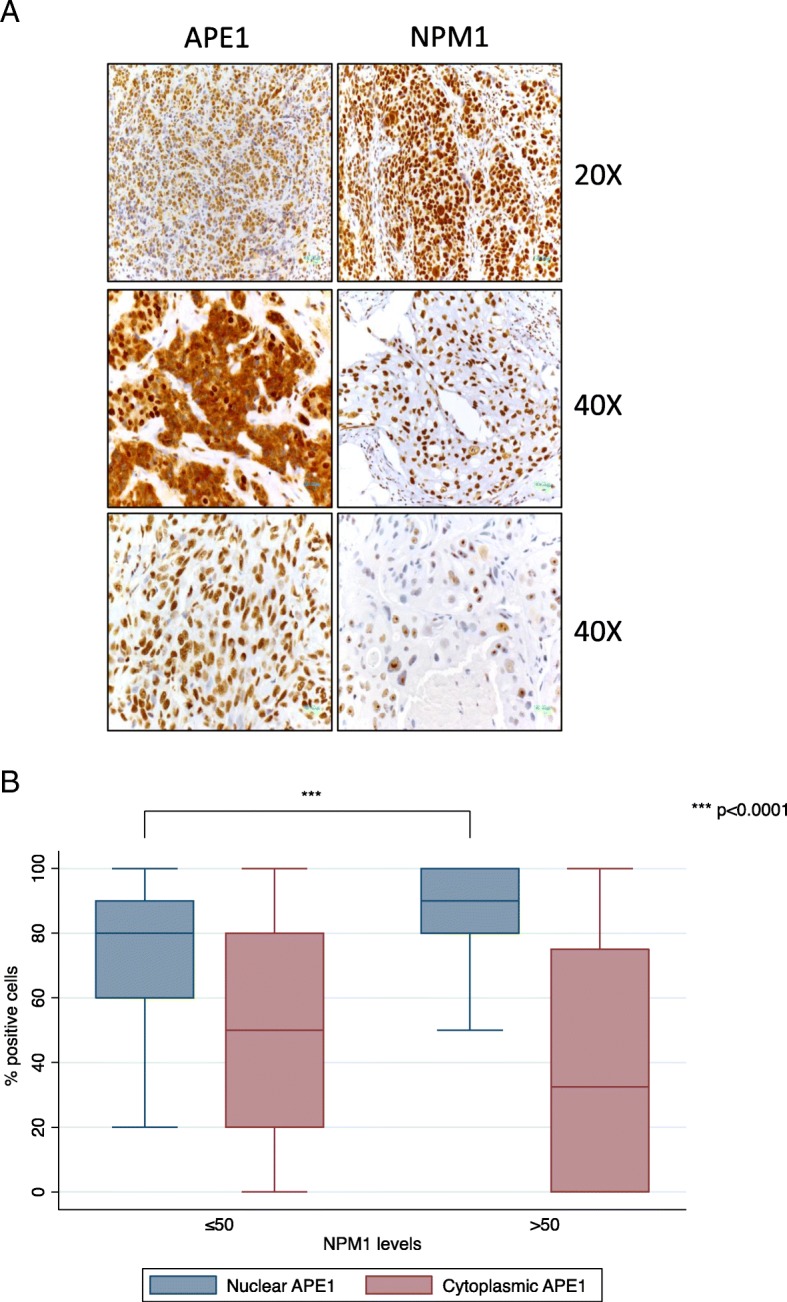
NPM1 IHC expression is associated with APE1 localization. Representative IHC samples of TNBC tumors, obtained at 20X and 40X magnification, revealing levels and localization of NPM1 and APE1 (**a**). Relative graph of nuclear vs cytoplasmic APE1 levels according to NPM1 (**b**). *** p < 0.0001 using the Mann-Whitney U test

Based on the observed significant relationship between APE1 and NPM1 in TNBC cell lines and their protective function towards Pt-compounds, we then checked the expression levels of both APE1 and NPM1 proteins in a real cohort of TNBC samples. Among the total population of 111 consecutive TNBC patients, 7 had de novo metastatic disease, while distant relapse was experienced by 27 patients. Median follow-up was 64 months [range 6;168]. In non-de novo metastatic patients, estimate OS at 12 and 60 months was 98 and 76%. Cumulative incidence at 12 and 60 months was 3.8 and 17.5%, respectively. Further clinico-pathological characteristics are summarized in Table 2. Expression levels and localization of the NPM1 and APE1 proteins were analyzed through IHC, as described in the Methodological section (Fig. 9a). Levels and localization of NPM1 were not associated with the presence of nodal (*p* = 0.588) or distant metastasis at baseline (*p* = 0.104). On the other hand, significantly lower levels of total NPM1 were found in tumors with a pT3 or pT4 stage (*p* = 0.046) and among patients who developed distant metastases during their whole clinical history (*p* = 0.011). Levels and localization of APE1 and NPM1 were then analyzed in order to explore potential in vivo correlations. NPM1 levels were significantly higher (*p* < 0.001) when the nucleolar localization of NPM1 was absent, a similar trend being observed also for the nuclear levels of APE1 (nAPE1) (*p* = 0.069). Higher levels of NPM1 were significantly correlated with higher levels of nAPE1 (p < 0.001), but not with cytoplasmic APE1 (cAPE1) (*p* = 0.119). On the other hand, high levels of cAPE1 were significantly correlated with lower levels of the nuclear counterpart (p < 0.001). A differential association between APE1 localization and NPM1 levels was also observed when dichotomization at 50% was applied for NPM1 (Fig. 9b).

**Table 2:** Demographic characteristics of the study populations. IQR: interquartile range; AR: androgen receptor nAPE1: nuclear APE1; cAPE1: cytoplasmic APE1

Categorical variables		
Variable (N)		Frequency (%)
Grading (99)	1	1 (1%)
	2	24 (24%)
	3	74 (75%)
Tumor size (92)	1	59 (64%)
	2	27 (29%)
	3 or 4	6 (7%)
Nodal status (91)	Positive	39 (57%)
	Negative	52 (43%)
De-novo metastatic (111)	Yes	7 (6%)
	No	104 (94%)
Neo/Adj Anthracyclines (104)	Yes	74 (71%)
	No	30 (29%)
Neo/Adj Taxanes (104)	Yes	63 (61%)
	No	41 (39%)
Continuous variables		
Variable (N)	Median	IQR
Age at diagnosis (111)	57	38–83
Ki67 (84)	70	2–98
AR (111)	0	0–90
nAPE1 (111)	90	70–100
cAPE1 (111)	40	2.5–80
NPM1 (111)	70	40–90

### NPM1 is associated with EFI in anthracycline-naïve patients

**Fig. 10:**
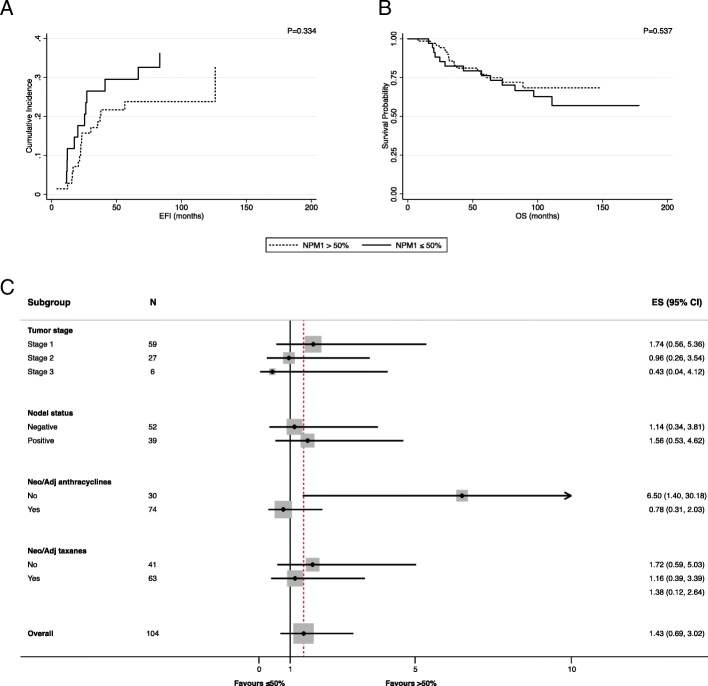
Prognostic role of NPM1 in TNBC. Impact of NPM1 on the whole TNBC population using a 50% cutoff showed as Cumulative Incidence (EFI) and Survival Probability (OS) (**a, b**). NPM1 subgroup analysis in terms of EFI according to tumor stage, nodal status and use of anthracyclines or taxanes in adjuvant or neoadjuvant setting (**c**)

The role of NPM1 was further explored with respect to prognosis. In the whole population, no associations were observed in terms of EFI (SHR: 1.43, 95% C.I. 0.69–3.02, *p* = 0.334). Consistent results were observed in terms of OS (Table 3) (Fig. 10a, b). A subgroup analysis was then performed in terms of EFI (Fig. 10c) and OS (data not shown) for hypothesis generation. Notably, in patients that were not treated with anthracyclines, low levels of NPM1 were associated with a worse prognosis in terms of EFI (SHR: 6.5, 95% C.I. 1.4–30.18, *p* = 0.017), but not of OS (data not shown).

**Table 3:** Univariate analysis in terms of OS (overall survival) and EFI (event free survival) with correction for competing risks and on the total population

Variable		SHR	95% C.I.	*p* value
EFI				
NPM1	Continuous	0.99	0.98–1.00	0.147
NPM1 nucleolus	No	1		
	Yes	1.83	0.79–4.27	0.161
NPM1 with cut-off	> 50%	1		
	≤50%	1.43	0.69–3.02	0.334
nAPE1	Continuous	0.99	0.98–1.00	0.344
cAPE1	Continuous	1.01	0.99–1.02	0.149
Tumor size	1	1		
	2	1.35	0.58–3.14	0.491
	3 or 4	3.48	0.79–15.44	0.100
Nodal status	Negative	1		
	Positive	2.05	0.92–4.58	0.079
Neo/Adj Anthracyclines	No	1		
	Yes	1.06	0.47–2.39	0.886
Neo/Adj Taxanes	No	1		
	Yes	0.82	0.39–1.69	0.585
OS		HR	95% C.I.	*p* value
NPM1 nucleolus	No	1		
	Yes	1.37	0.59–3.20	0.464
NPM1 with cut-off	> 50%	1		
	≤50%	1.25	0.61–2.57	0.541
nAPE1	Continuous	1	0.99–1.01	0.767
cAPE1	Continuous	1	0.99–1.01	0.553
Tumor size	1	1		
	2	1.73	0.73–4.11	0.214
	3 or 4	12.59	4.30–36.84	< 0.001
Nodal status	Negative	1		
	Positive	1.47	0.68–3.17	0.328
Neo/Adj Anthracyclines	No	1		
	Yes	0.45	0.22–0.91	0.026
Neo/Adj Taxanes	No	1		
	Yes	0.50	0.24–1.02	0.055

Tumor size was confirmed as the most important prognostic factor. nAPE1 (nuclear APE1), cAPE1 (cytoplasmic APE1) NPM1 (NPM1^low^ vs NPM1^high^), HR (hazard ratio), SHR (subdistribution hazard ratio)

**Table 4:** Detailed description of TCGA tumor datasets having a statistically significant APE1/NPM1 gene expression correlation. Every cohort is represented by its symbol (as shown in Additional file 6: Figure S5), full description, RNA-seq gene expression correlation between APE1 and NPM1 and statistical significance of the data

Cohort	Description	Corr	P value
PRAD	Prostate adenocarcinoma	0.644	1.58E-59
KICH	Kidney Chromophobe	0.590	1.88E-07
PAAD	Pancreatic adenocarcinoma	0.517	1.41E-13
SARC	Sarcoma	0.503	5.22E-18
GBMLGG	Glioma	0.501	9.73E-44
THYM	Thymoma	0.462	1.07E-07
LUAD	Lung adenocarcinoma	0.448	8.68E-27
THCA	Thyroid carcinoma	0.445	1.00E-25
KIPAN	Pan-kidney cohort (KICH+KIRC+KIRP)	0.431	1.30E-41
KIRP	Kidney renal papillary cell carcinoma	0.431	1.48E-14
STES	Stomach and Esophageal carcinoma	0.415	2.24E-26
STAD	Stomach adenocarcinoma	0.412	1.77E-18
ESCA	Esophageal carcinoma	0.398	2.28E-08
KIRC	Kidney renal clear cell carcinoma	0.392	5.52E-21
CESC	Cervical squamous cell carcinoma and endocervical adenocarcinoma	0.380	7.13E-12
UCEC	Uterine Corpus Endometrial Carcinoma	0.369	4.73E-19
LIHC	Liver hepatocellular carcinoma	0.348	5.09E-12
COAD	Colon adenocarcinoma	0.344	3.87E-14
GBM	Glioblastoma multiforme	0.306	1.17E-04
COADREAD	Colorectal adenocarcinoma	0.291	1.25E-13
BRCA	Breast invasive carcinoma	0.282	1.74E-21
SKCM	Skin Cutaneous Melanoma	0.270	5.76E-03
HNSC	Head and Neck squamous cell carcinoma	0.264	1.04E-09
LUSC	Lung squamous cell carcinoma	0.246	2.31E-08
BLCA	Bladder Urothelial Carcinoma	0.244	6.14E-07

### Identification of APE1/NPM1-related gene signatures in TCGA-BRCA tumors

**Fig. 11:**
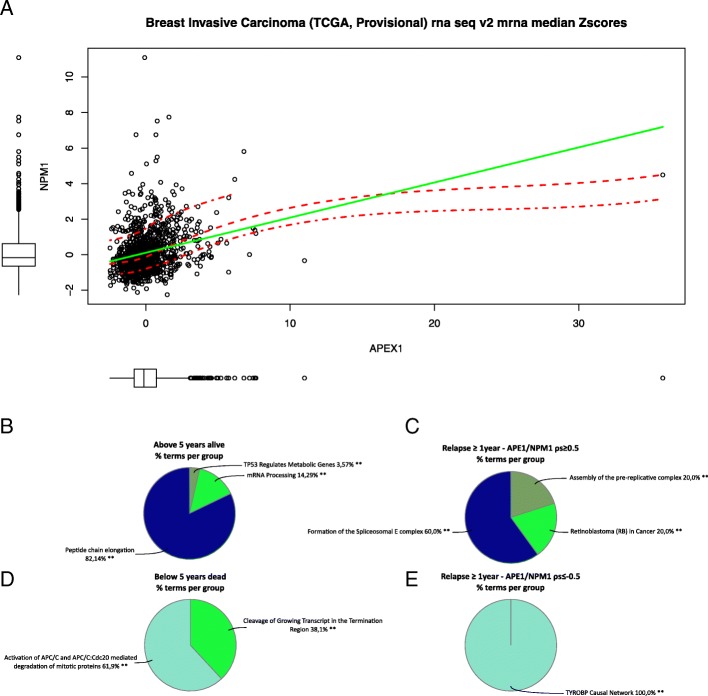
Comparison of APE1 and NPM1 gene expression levels and functional characterization of APE1/NPM1 correlated genes. Scatter plot showing the relationship existing between APE1 and NPM1 expression levels in TCGA Breast Invasive Carcinoma patients (*n* = 1105). The red dash-dotted lines represent the first and third quartile fits, the central dashed line represents the mean smooth and the green line represents the fitted regression line (Ordinary Least Squares). Marginal boxplots are also shown (**a**). Two lists of genes with APE1/NPM1 correlated expression (ρs ≥ + 0.5 or ρs ≤ − 0.5, *p* ≤ 0.05) were identified in two groups of TNBC patients having an OS higher (**b**, *n* = 44) or lower (**c**, *n* = 23) than 5 years. Functionally enriched terms (Benjamini-Hochberg adjusted p ≤ 0.05) were identified using the Cytoscape plugin ClueGO, querying several functional and metabolic databases, and results were summarized as pie charts. The same analysis was repeated on a group of patients (n = 23) that had a relapse of the disease after at least one year from diagnosis, analyzing separately the gene signatures with positive (ρs ≥ + 0.5, p ≤ 0.05, *n* = 54) or negative (ρs ≤ − 0.5, p ≤ 0.05, *n* = 61) correlation with APE1/NPM1 (**d, e**)

In order to generalize our findings and to investigate the APE1 and NPM1 overall influence and association with specific gene-signatures on tumorigenesis, we then followed a more global approach using the available cancer datasets from TCGA. Using the cgdsr R/Bioconductor package, we analyzed all the TCGA datasets associated with RNA-seq data (*n* = 36) and measured, on a *per sample* basis, the correlation existing between APE1 and NPM1 gene expression, represented by the mRNA median z-scores of every patient (Additional file 6: Figure S5). Out of twenty-five datasets (Table 4), in which a significant correlation was obtained, Prostate Adenocarcinoma and Bladder Urothelial Carcinomas were the two having the highest and the lowest values (*p* = 0.64 and *p* = 0.24, respectively), with Breast Invasive Carcinoma showing a quite weak correlation (*p* = 0.28, Fig. 11a). We then wondered if APE1 and NPM1 could indirectly affect disease recurrence and overall survival, by acting through the involvement of co-expressed genes that could be functionally associated with tumorigenesis. For this reason, we specifically examined TCGA-BRCA TNBC patients, focusing on three different subsets: patients with an OS lower (*n* = 23) or higher (*n* = 44) than five years, and patients that experienced tumor recurrence after at least one year from diagnosis (n = 23). For each group of patients, we first defined the genes having positive (ρs ≥ + 0.5, *p* ≤ 0.05) or negative correlation (ρs ≤ − 0.5, p ≤ 0.05) with either APE1 or NPM1 gene expression and, afterwards, we looked for co-occurrences between the different gene lists. Surprisingly, all the common genes were always positively or negatively correlated with both APE1 and NPM1. In particular, APE1/NPM1 showed positive or negative correlation with twenty-four and eleven genes, respectively, in patients having OS ≥5 years, and with thirty and seven genes, respectively, in patients having OS < 5 years (Additional file 7: Figure S6A). Moreover, patients experiencing tumor recurrence, after at least one year from diagnosis, had fifty-four genes that were positively correlated with APE1/NPM1, while sixty-one had negative correlation (Additional file 7: Figure S6B). An additional Excel file contains the complete lists of positively and negatively APE1/NPM1 correlated genes [see Additional file 1]. Finally, these signatures were functionally characterized using the Cytoscape plugin ClueGO, querying several functional and metabolic databases. Among the significantly enriched results (adjusted p ≤ 0.05), we found terms pointing to mRNA processing and peptide chain elongation (Fig. 11b-e), processes that could be the targets of new precision medicine approaches.

## Discussion

Pt-salts represent a common chemotherapeutic option for the treatment of several tumors, including breast cancer. Based on the evidences regarding the role of BER factors in regulating Pt-salts cytotoxicity, and in order to shed more light on the molecular basis of BER protective function to Pt-compounds treatment, the present study was aimed at characterizing the functional relationship between APE1 and NPM1, as well as their interaction with Pt-compounds. We focused specifically on TNBC, a particularly aggressive tumor subtype, characterized by poor prognosis and lack of novel therapeutic targets [11, 84], in which, currently, genotoxic agents are gaining momentum as an important option [15]. The genotoxic effect of Pt-salts, generating bulky lesions on DNA, can be efficiently opposed by different DNA repair pathways, including NER, HR and FA. Only recently, BER has emerged as a putative DNA repair pathway involved in cancer cell resistance to Pt-salts treatments. However, the way in which BER is involved still remains object of study. It has been demonstrated that Pol β [85] could be considered a prognostic factor in colorectal and esophageal cancer, for its ability to affect Pt-salts susceptibility [63, 86, 87]. Similarly, APE1, which is overexpressed in lung cancer, is directly involved in CDDP resistance and its inhibition sensitizes cancer cells to the treatment [68]. Conflicting observations were reported by Kothandapani et al., which have hypothesized that BER could play a role in mediating the CDDP cytotoxicity, showing how a BER blockage, through Pol β or UNG1 knockout or treatment with Methoxyamine, an indirect inhibitor of APE1 endonuclease activity, may result in an increased resistance to CDDP treatment [20, 21]. What is actually unclear is why BER could be directly involved in the CDDP- induced DNA damage, since it is not able to process any bulky DNA distortion produced by Pt-induced adducts. Interestingly, several authors have hypothesized an indirect involvement of BER in mediating cisplatin cytotoxicity [64, 65]. Again, Kothandapani et al. showed that CDDP-treatment induces abasic sites accumulation close to ICLs [20]. It also seems that CDDP and, to a lesser extent, CBDCA and oxaliplatin may cause the generation of reactive oxygen species (ROS), significantly increasing oxidative stress levels. Moreover, interesting findings have recently shown how Pt-salts may increment ROS and DNA oxidation, leading to an increased cell death [64], and have suggested a possible use of APE1 redox inhibitors for the treatment/prevention of secondary effects like Pt-induced sensory neuropathy [88]. A further hypothesis regards the emerging discoveries about the non-canonical roles of BER proteins such as those associated with RNA metabolism [23], that could also explain the role of BER in Pt-salts induced effects. In fact, a strong but not completely characterized association between Pt-salts and RNA-damage, which inhibits ribosomal RNA synthesis in vivo [89] and mRNA translation in vitro [90], has been found.

In this study, by using APE1- or NPM1-defective isogenic cell lines, we first demonstrated a leading role of APE1 and NPM1 expression, as well as their functional interaction, accounting for cell resistance to CDDP cytotoxicity. Then, by using two TNBC cell lines (HCC70 and HCC1937 cells) characterized by a different p53- and BRCA1-status, we initially categorized their response to Pt-salts (CDDP and CBDCA) in vitro. Specifically, we observed that HCC70 cells, having lower levels of both APE1 and NPM1 proteins, were more sensitive to Pt-salts treatments compared to HCC1937 cells; in particular, we found that this occurred in a NPM1-dependent manner, as demonstrated by the protective effect due to the overexpression of NPM1 in the HCC70 cell line. In order to evaluate whether APE1 was also involved in the response to Pt-salts in TNBC cells, we tested the effect of the combination of CDDP and CBDCA with different APE1 inhibitors targeting the endonuclease activity of the protein (e.g. Compound #3) and its interaction with NPM1 (Fiduxosin, Spiclomazine and SB206553) [59, 61]. After evaluating the response of both TNBC cell lines to these drugs as single agents, which demonstrated that the HCC70 cell line was more sensitive to APE1-inibitors, in agreement with the lower level of the protein with respect to HCC1937, we analyzed the response of the combined treatment with Pt-salts. Interestingly, we observed that only HCC70 cells, expressing lower levels of both APE1 and NPM1, were sensitized by the combination of Compound #3 or Fiduxosin with both crosslinking agents. We previously reported that APE1 accumulates within the nucleoli through the interaction with NPM1 [28]. Notably, in that paper we observed that CDDP induced a re-localization of both NPM1 and APE1 to the nucleoplasm in HeLa cells and that impairment of the nucleolar-nucleoplasmic shuttling kinetics of APE1 was associated with a higher sensitivity to CDDP induced cytotoxicity [43]. Our present data in TNBC cell lines, showing nucleolar emptying upon Pt-salts treatment but having different kinetics between HCC70 and HCC1937 cell lines, allow to conclude that APE1 and NPM1 proteins re-localization from nucleoli to nucleoplasm after treatment with Pt-compounds is a general phenomenon of cancer cells, even though with different kinetics. The molecular mechanisms at the basis of this phenomenon should be better clarified, as well as their biological significance and relevance for cancer therapeutics, and work is actually ongoing in our laboratory along these lines. In order to explore the translational and clinical relevance of our results, we explored the mutual interactions of APE1 and NPM1 and their associations with clinico-pathological features in a real-world cohort of TNBC patients. Both APE1 and NPM1 biomarkers were analyzed using a healthcare-grade analytical pipeline, without any ad-hoc adaptations, rendering this approach scalable and integrable with the current clinical workflow. Moreover, expression level correlations and localization associations were consistent with those observed in our preclinical models. Notably, APE1 was not associated with any common clinical characteristics, while NPM1 was associated with worse disease onset and interesting results were observed in the exploratory outcome analysis, in terms of both protein levels and localization. On the other hand, NPM1 failed to show a prognostic role for the total population; however, the subgroup analysis highlighted a potential role in terms of EFI among patients not treated with anthracyclines in the neoadjuvant or adjuvant setting, suggesting that anthracycline naïve patients with low NPM1 have a significantly higher probability of having a local or distant relapse. Since NPM1 has a pivotal role in controlling ribosomal biogenesis and genome stability [91], these data are an intriguing hint in refining patients’ stratification according to potential response to genotoxic agents. As the anthracycline-based regimen seems to bridge the gap between the NPM1 low and high groups, these data suggest that there is a subgroup of TNBC patients that not only is potentially prone to respond, but that could be burdened by a worse outcome if different therapeutic strategies were put in place. These results are of particular clinical interest since DNA-damaging strategies are an emerging opportunity for the treatment of TNBC, but a solid predictive marker is currently needed [12, 17, 92]. The inability to translate this difference on OS could be due to the small sample size, post-relapse treatments or underlying differences in terms of tumor biology. In order to further elucidate this aspect, we investigated the overall impact of APE1 and NPM1 levels through TCGA-derived RNA-seq data. To find promising companion proteins, based on the hints derived by our clinical results, we then compared patients with an OS lower or higher than 5 years, and those with a recurrence after at least 1 year from diagnosis and we identified subsets of genes (Additional file 7: Figure S6) that positively or negatively correlated with APE1 and NPM1 levels. Interestingly, the functional terms associated with these signatures highlighted biological processes that could represent new hubs for personalized anti-cancer treatments. We found of particular interest the alterations concerning cell-cycle dependent proteins observed among patients with an OS < 5 years. Targeting cell cycle is a new emerging strategy that was particularly effective in luminal-like breast cancer in reverting endocrine resistance [93], but interesting data suggest a possible role of CDK4–6 inhibitors also as chemo-companions, since CDK6 may interfere with Pt-induced cell death through FOXO3 [94]. The present study, therefore, supports the rationale of finding optimal DNA-repair - focused companions, capable to both synergize with chemotherapy and to revert potential resistance mechanisms that could defeat the DNA - damaging potential of Pt-salts [12]. Synthetic lethality involving the inhibition of the APE1 endonuclease activity is only recently emerging in literature [95, 96]. The differential response of the TNBC cell lines here tested is certainly interesting, as it could be helpful in understanding TNBC heterogeneity and consequently tailor the right therapeutic associations and sequences on the single patient. This possibility is currently under investigations in our laboratory, but it is likely that the different mutational status of p53 could have a role in the observed differences. As reported in the Materials and Methods section, the mutational status of *TP53* is different among the two TNBC cell lines used in the present study. The R^248^Q mutation, occurring in HCC70 cells, represents a “hot spot” mutation in the DNA binding domain (DBD) of the transcription factor (amino acids 109–288), often found in several types of tumors. This mutation belongs to the class of mutagenic p53 that affects the DNA binding activity without destabilizing the DBD [97, 98]. Generally, R^248^Q is a functional mutation of p53 that causes an aberrant overexpression of the p53 protein [99]. Just recently, Olszewski et al. have demonstrated that the R^248^ mutational status may affect cancer cell biology, in a cell-type dependent manner, demonstrating how in breast cancer the expression of R^248^Q p53 decreases the motility and invasiveness [100], differently from what has been published in other tumors types [101, 102]. Contrarily to the HCC70 cell line, the HCC1937 cells have an acquired non-sense mutation (C^306^T) occurring on the *TP53* gene and giving rise to a conversion of a codon encoding for R^306^ to a STOP codon, which occurs close to the tetramerization domain (amino acids 324–359) and thus causes a negative expression of p53 [103]. Data not shown from our laboratory (Codrich et al., submitted) clearly demonstrated that the inhibition of the APE1 endonuclease activity by Compound #3, in different cancer cell lines including HCC70 and HCC1937, causes a reduction of cell viability in a p53-dependent manner, possibly involving nucleolar stress and impairment of ribosome biogenesis [43]. Based on these premises, the lack of sensitization response of HCC1937 cells to the combined-treatment with Pt-compounds and Compound #3 could indeed be linked to a potential differential effect mediated by the p53 functional status.

In the case of BRCA1 gene, which is important for correct functional activity of HR in repairing DSBs formation generated by Pt-compounds, it was somehow surprising that HCC1937 cells (BRCA1-deficient), see Materials and Methods section for details, were more resistant to cisplatin than HCC70 cells (BRCA1-proficient). We believe that, in the time-frame and doses we considered in our experimental set up, the effect on cell viability was not only due to a difference in the DNA repair activity by HR, by means of BRCA1 functional protein, but also to damages associated to RNA processing mechanisms, which are correlated with APE1/NPM1 functional activities. Moreover, a major role of NHEJ in repairing DSBs induced by Pt-compounds treatment by HCC1937 cell line, cannot be excluded at present. Thus, further studies are needed to drive definitive conclusions on these hypotheses.

## Conclusion

The present study analyzed the relevance of APE1, NPM1 and their interaction towards Pt-salts cell cytotoxicity and explored their role as clinically transferable biomarkers for patients’ selection and hypothesis generation. APE1 and NPM1 protect cancer cells from Pt-compounds cytotoxicity, suggesting a possible improvement of the activity of Pt-based therapy for TNBC, using the NPM1 and APE1 proteins as second therapeutic targets. We propose several TNBC gene signatures having positive or negative correlation with APE1 and NPM1. Of particular interest, APE1/NPM1 gene expression levels are associated with the alterations concerning cell-cycle dependent proteins observed among patients with an OS < 5 years.

## Additional files

Additional file 1: Complete lists of negatively and positively APE1/NPM1 correlated genes. Sheet A. List of APE1 (col. A-C) and NPM1 (col. E-G) negatively or positively correlated genes (ρs ≤ − 0.2 or ρs ≥ + 0.2, respectively) in TCGA-BRCA patients with OS ≥5 years (*n* = 44). Columns I-L contain the subset of genes having ρs ≤ − 0.5 or ρs ≥ + 0.5, *p* ≤ 0.05 that underwent the functional analysis phase. Sheet B. Same as A, but for patients that died after 5 or more years (*n* = 5). Sheet C. Same as A, but for patients that were censored within 5 years (*n* = 108). Sheet D. Same as A, but for patients with OS < 5 years (*n* = 23). Sheet E. Recapitulative lists of genes from Sheet A and D that underwent the functional analysis phase. Sheet F. Same as A, but for patients that experienced tumor recurrence after at least one year from diagnosis (n = 23). (DOCX 26 kb)

Additional file 2: **Figure S1.** CDDP induces a nucleolar emptying of APE1 and a nucleoplasmic re - localization of NPM1. (**a**, **b**) Immunofluorescence staining for APE1 on HCC70 (**a**) and HCC1937 (**b**) cells shows an evident nucleolar emptying of APE1 upon CDDP treatment (3.125 μM) in a time-dependent trend. The nucleolar repopulation is observed in HCC70 cells only, after 24 h of treatment. HCC1937 cells responded differently, with a prolonged emptying of nucleolar APE1 that persisted up to 72h of treatment. (**c**, **d**)Immunofluorescence staining for NPM1 on HCC70 (**c**)and HCC1937 (**d**) cells shows an increased accumulation of NPM1 into the nucleoplasmic compartment upon CDDP treatment (3.125 μM) in a time-dependent trend. Yellow arrowheads highlight representative cells showing the characteristic phenotype as described. ‘Untreated’ corresponds to the condition in which the cells didn’t undergo any treatment at the corresponding longer point of time. TOPRO-3 staining was used to identify nuclei. ‘Merge’ indicates the overlapping of the signals of APE1 (or NPM1) and TOPRO-3. (PDF 968 kb)

Additional file 3: **Figure S2.** CBDCA induces a nucleolar emptying of APE1 and a nucleoplasmic re - localization of NPM1. (**a**, **b**)Immunofluorescence staining for APE1 on HCC70 upon CBDCA treatment (100 μM)(**a**) and HCC1937 upon CBDCA treatment (25 μM) (**b**)cells shows an evident nucleolar emptying of APE1 in a time-dependent trend. The nucleolar repopulation is observed in HCC70 cells only, after 24 h of treatment. HCC1937 cells responded differently, with a prolonged emptying of nucleolar APE1 that persisted up to 72h of treatment. (**c**, **d**) Immunofluorescence staining for NPM1 on HCC70 upon CBDCA treatment (100 μM) (**c**)and HCC1937 upon CBDCA treatment (25 μM) (**d**) cells shows an increased accumulation of NPM1 into the nucleoplasmic compartment in a time-dependent trend. Yellow arrowheads highlight representative cells showing the characteristic phenotype as described. ‘Untreated’ corresponds to the condition in which the cells didn’t undergo any treatment at the corresponding longer point of time. TOPRO-3 staining was used to identify nuclei. ‘Merge’ indicates the overlapping of the signals of APE1 (or NPM1) and TOPRO-3. (PDF 999 kb)

Additional file 4: **Figure S3**. Chronic treatment with Pt-compounds induces an increase of NPM1 on HCC70 cells. (**a**) *(up)* Representative western blotting shows the NPM1 protein levels trend in HCC70 cells treated with CDDP at different time points (*right*) or different concentrations (*left*), as specified on the top of the panel. On the right side of each panel, the Molecular Weights (*MW*), expressed in *kDa*, are indicated. On the left side of each panel, specific antibodies, used in the immunoblotting, are indicated. *(bottom)* Histograms reporting the quantitative values corresponding to the NPM1 protein amounts compared to the basal untreated conditions and normalized on Tubulin. Values express the mean viability ± SD from at least three independent replicates. *p < 0.05. (**b**) *(up)* Representative western blotting shows the NPM1 protein levels in HCC70 cells, treated with CBDCA at different concentrations or different time points, as specified on the top of the panel. On the right side of each panel, the Molecular Weights (*MW*), expressed in *kDa*, are indicated. On the left side of each panel, specific antibodies, used in the immunoblotting, are indicated. *(bottom)* Histograms reporting the quantitative values corresponding to the NPM1 protein amounts compared to the basal untreated conditions and normalized on Tubulin. Values express the mean viability ± SD from at least three independent replicates. *p < 0.05. (PDF 587 kb)

Additional file 5: **Figure S4.** Spiclomazine inhibitor does not sensitize HCC70 cells to Pt-compounds treatment. Histograms show the effect of co-treatments with CDDP (**a**, **d**, **f**) or CBDCA (**b**, **c**, **e**, **g**) and APE1/NPM1 inhibitors, Spiclomazine (**a**, **b**, **d**, **e**) and SB206553 (**c**, **f**, **g**)on HCC70 cells viability. Values express the mean viability ± SD from at least three independent replicates. Each value is normalized to the untreated condition. (PDF 81 kb)

Additional file 6: **Figure S5**. Comparison of APE1 and NPM1 gene expression levels in TCGA tumor datasets. Barplot summarizing the statistically significant correlations (*p* ≤ 0.05) existing between APE1 and NPM1 expression levels in twenty-five TCGA tumor samples. RNA-seq data for thirty-six TCGA datasets were extracted and Pearson correlation coefficients between APE1 and NPM1 were calculated on a per sample basis, retaining and plotting only the statistically significant results. The number of patients profiled in every dataset is shown on top of each bar; bar colors reflect the size of the correlations. (PDF 96 kb)

Additional file 7: **Figure S6.** Venn diagrams showing similarities and differences existing among the examined gene lists. Differences in patients’ overall survival below or above 5 years allowed to identify four gene signatures that positively or negatively correlated with both APE1 and NPM1 gene expression (**a**). Differences in disease recurrence after at least one year from diagnosis defined several specific signatures as well as two lists of genes that had a positive or negative correlation with both APE1 and NPM1 gene expression (**b**). (PDF 105 kb)

## Data Availability

The datasets generated and analyzed during the current study are not publicly available due to relevant data protection laws. The data may be available upon reasonable request to the corresponding author. TCGA data is publicly available.

## Abbreviations

APE1: Apurinic/apyrimidinic endonuclease 1
BC: Breast cancer
BER: Base excision repair
CBDCA: Carboplatin
CDDP: Cisplatin
NPM1: Nucleophosmin
Pt: Platinum
TNBC: Triple negative breast cancer
