# Supplementary material for: APE1 and NPM1 protect cancer cells from platinum compounds cytotoxicity and their expression pattern has a prognostic value in TNBC
Source: J Exp Clin Cancer Res. 2019 Jul 15;38:309. doi: 10.1186/s13046-019-1294-9 (PMC6631760; doi:10.1186/s13046-019-1294-9)
Supplement: Supplementary file 1 — Complete lists of negatively and positively APE1/NPM1 correlated genes. Sheet A. List of APE1 (col. A-C) and NPM1 (col. E-G) negatively or positively correlated genes (ρs ≤ − 0.2 or ρs ≥ + 0.2, respectively) in TCGA-BRCA patients with OS ≥5 years (n = 44). Columns I-L contain the subset of genes having ρs ≤ − 0.5 or ρs ≥ + 0.5, p ≤ 0.05 that underwent the functional analysis phase. Sheet B. Same as A, but for patients that died after 5 or more years (n = 5). Sheet C. Same as A, but for patients that were censored within 5 years (n = 108). Sheet D. Same as A, but for patients with OS < 5 years (n = 23). Sheet E. Recapitulative lists of genes from Sheet A and D that underwent the functional analysis phase. Sheet F. Same as A, but for patients that experienced tumor recurrence after at least one year from diagnosis (n = 23). (DOCX 26 kb) [file 13046_2019_1294_MOESM1_ESM.docx]

**APE1 and NPM1 protect cancer cells from platinum compounds cytotoxicity and their expression pattern has a prognostic value in TNBC**

Matilde Clarissa Malfatti^1^, Lorenzo Gerratana^1,2^, Emiliano Dalla^1^, Miriam Isola^1^, Giuseppe Damante^1^, Carla Di Loreto^1,3^, Fabio Puglisi^1,4^ and Gianluca Tell^1, *^

^1^ Department of Medicine (DAME), University of Udine, Udine, Italy.

^2^ Department of Oncology, ASUI Udine SMM University Hospital Udine, Italy.

^3^ Department of Pathology, ASUI Udine SMM University Hospital Udine, Italy.

^4^ Department of Medical Oncology, Centro di Riferimento Oncologico (CRO), IRCCS, Aviano, Italy

*Corresponding Author: Prof. Gianluca Tell,

Head of the Laboratory of Molecular Biology and DNA repair

Department of Medicine

University of Udine

Piazzale M. Kolbe 4

33100 Udine – Italy

[gianluca.tell@uniud.it](mailto:gianluca.tell@uniud.it)

[Tel:+39](tel:+39) 0432 494311

Fax: +39 0432 494301

**SUPPLEMENTAL INFORMATIONS**

**Supplementary Figure Legends**

**Supplementary Figure S1.** CDDP induces a nucleolar emptying of APE1 and a nucleoplasmic re - localization of NPM1. **(A-B)** Immunofluorescence staining for APE1 on HCC70 **(A)** and HCC1937 **(B)** cells shows an evident nucleolar emptying of APE1 upon CDDP treatment (3.125 μM) in a time-dependent trend. The nucleolar repopulation is observed in HCC70 cells only, after 24 hours treatment. HCC1937 cells responded differently, with a prolonged emptying of nucleolar APE1 that persisted up to 72-hours of treatment. **(C-D)** Immunofluorescence staining for NPM1 on HCC70 **(C)** and HCC1937 **(D)** cells shows an increased accumulation of NPM1 into the nucleoplasmic compartment upon CDDP treatment (3.125 μM) in a time-dependent trend. Yellow arrowheads highlight representative cells showing the characteristic phenotype as described. ‘Untreated’ corresponds to the condition in which the cells didn’t undergo any treatment at the corresponding longer point of time. TOPRO-3 staining was used to identify nuclei. ‘Merge’ indicates the overlapping of the signals of APE1 (or NPM1) and TOPRO-3.

**Supplementary Figure S2.** CBDCA induces a nucleolar emptying of APE1 and a nucleoplasmic re - localization of NPM1. **(A-B)** Immunofluorescence staining for APE1 on HCC70 upon CBDCA treatment (100 μM) **(A)** and HCC1937 upon CBDCA treatment (25 μM) **(B)** cells shows an evident nucleolar emptying of APE1 in a time-dependent trend. The nucleolar repopulation is observed in HCC70 cells only, after 24 hours treatment. HCC1937 cells responded differently, with a prolonged emptying of nucleolar APE1 that persisted up to 72-hours of treatment. **(C-D)** Immunofluorescence staining for NPM1 on HCC70 upon CBDCA treatment (100 μM) **(C)** and HCC1937 upon CBDCA treatment (25 μM) **(D)** cells shows an increased accumulation of NPM1 into the nucleoplasmic compartment in a time-dependent trend. Yellow arrowheads highlight representative cells showing the characteristic phenotype as described. ‘Untreated’ corresponds to the condition in which the cells didn’t undergo any treatment at the corresponding longer point of time. TOPRO-3 staining was used to identify nuclei. ‘Merge’ indicates the overlapping of the signals of APE1 (or NPM1) and TOPRO-3.

Supplementary Figure S3. Chronic treatment with Pt-compounds induces an increase of NPM1 on HCC70 cells. (A) *(up)* Representative western blotting shows the NPM1 protein levels trend in HCC70 cells treated with CDDP at different time points (*right*) or different concentrations (*left*), as specified on the top of the panel. On the right side of each panel, the Molecular Weights (*MW*), expressed in *kDa*, are indicated. On the left side of each panel, specific antibodies, used in the immunoblotting, are indicated. *(bottom)* Histograms reporting the quantitative values corresponding to the NPM1 protein amounts compared to the basal untreated conditions and normalized on Tubulin. Values express the mean viability ± SD from at least three independent replicates. *p<0.05. (B) *(up)* Representative western blotting shows the NPM1 protein levels in HCC70 cells, treated with CBDCA at different concentrations or different time points, as specified on the top of the panel. On the right side of each panel, the Molecular Weights (*MW*), expressed in *kDa*, are indicated. On the left side of each panel, specific antibodies, used in the immunoblotting, are indicated. *(bottom)* Histograms reporting the quantitative values corresponding to the NPM1 protein amounts compared to the basal untreated conditions and normalized on Tubulin. Values express the mean viability ± SD from at least three independent replicates. *p<0.05.

**Supplementary Figure S4.** **Spiclomazine inhibitor does not sensitize HCC70 cells to Pt- compounds treatment.** Histograms show the effect of co-treatments with CDDP **(A-D-F)** or CBDCA **(B-C-E-G)** and APE1/NPM1 inhibitors, Spiclomazine **(A-B-D-E)** and SB206553 **(C-F-G)** on HCC70 cells viability. Values express the mean viability ± SD from at least three independent replicates. Each value is normalized to the untreated condition.

**Supplementary Figure S5. Comparison of APE1 and NPM1 gene expression levels in TCGA tumor datasets.** Barplot summarizing the statistically significant correlations (P ≤ 0.05) existing between APE1 and NPM1 expression levels in twenty-five TCGA tumor samples. RNA-seq data for thirty-six TCGA datasets were extracted and Pearson correlation coefficients between APE1 and NPM1 were calculated on a per sample basis, retaining and plotting only the statistically significant results. The number of patients profiled in every dataset is shown on top of each bar; bar colors reflect the size of the correlations.

**Supplementary Figure S6.** Venn diagrams showing similarities and differences existing among the examined gene lists. Differences in patients’ overall survival below or above 5 years allowed to identify four gene signatures that positively or negatively correlated with both APE1 and NPM1 gene expression (**A**). Differences in disease recurrence after at least one year from diagnosis defined several specific signatures as well as two lists of genes that had a positive or negative correlation with both APE1 and NPM1 gene expression (**B**).

**Immunofluorescence analysis**

8,0 x 10^4^ cells were grown and treated on glass coverslips. Over the time of treatment, cells were fixed with 4% (wt/vol) PFA for 20 minutes, permeabilized with PBS–0.25% (vol/vol) Triton X-100 for 5 minutes and then blocked in 10% (vol/vol) FBS in TBS–0.1% (wt/vol) Tween-20 (blocking solution) for 30 minutes at RT. Following the incubation with primary antibodies [polyclonal α-APE1 (diluted 1:100, o/n, 4°C) and monoclonal α-NPM1 (diluted 1:200, o/n, 4 °C)] diluted in blocking solutions, cells were washed three times for 5 minutes each with TBS–0.1% (wt/vol) Tween-20 and then incubated with secondary antibody α-mouse Alexa Fluor 488-conjugated (diluted 1:400, 2 hours, RT) complemented with RNase A (diluted 1:100). After washing three times, coverslips were incubated with TO-PRO3 (diluted 1:500, 10 minutes, RT), washed and air dried. Finally, coverslips were mounted on microscope slides with a mounting media. Immunofluorescence images were acquired using a Leica TCS SP laser-scanning confocal microscope (Leica Microsystems, Wetzlar, Germany) equipped with a 488-nm argon laser, a 543-nm HeNe laser, and a 63X oil objective (HCX PL APO 63X Leica).
