# Supplementary figures and images for: APE1 and NPM1 protect cancer cells from platinum compounds cytotoxicity and their expression pattern has a prognostic value in TNBC
Source: J Exp Clin Cancer Res. 2019 Jul 15;38:309. doi: 10.1186/s13046-019-1294-9 (PMC6631760; doi:10.1186/s13046-019-1294-9)

Supplementary Figure S1

A

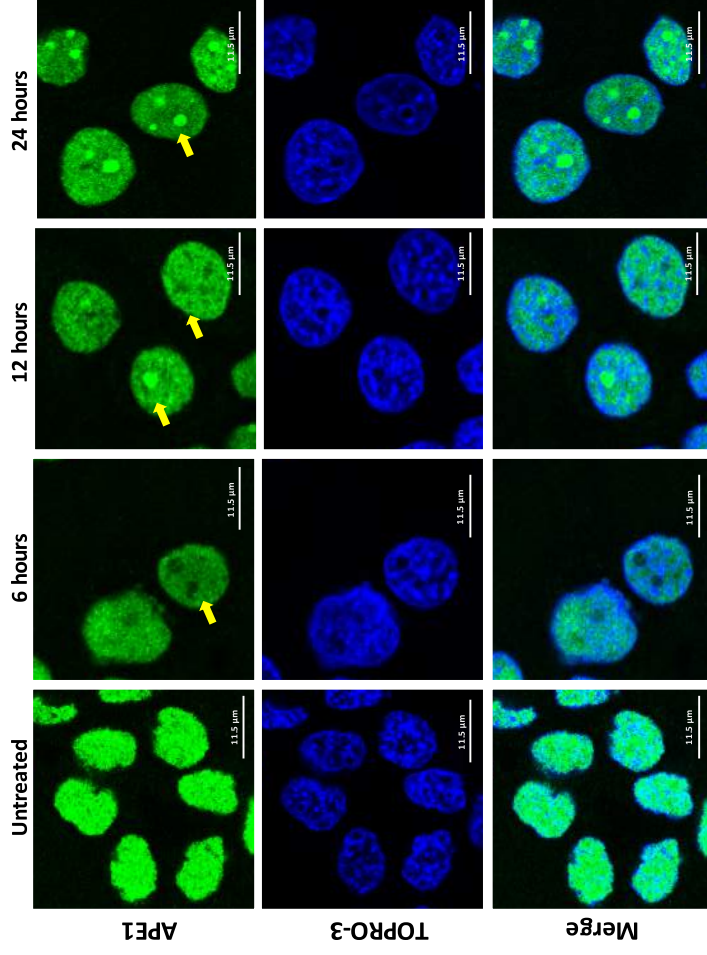

B

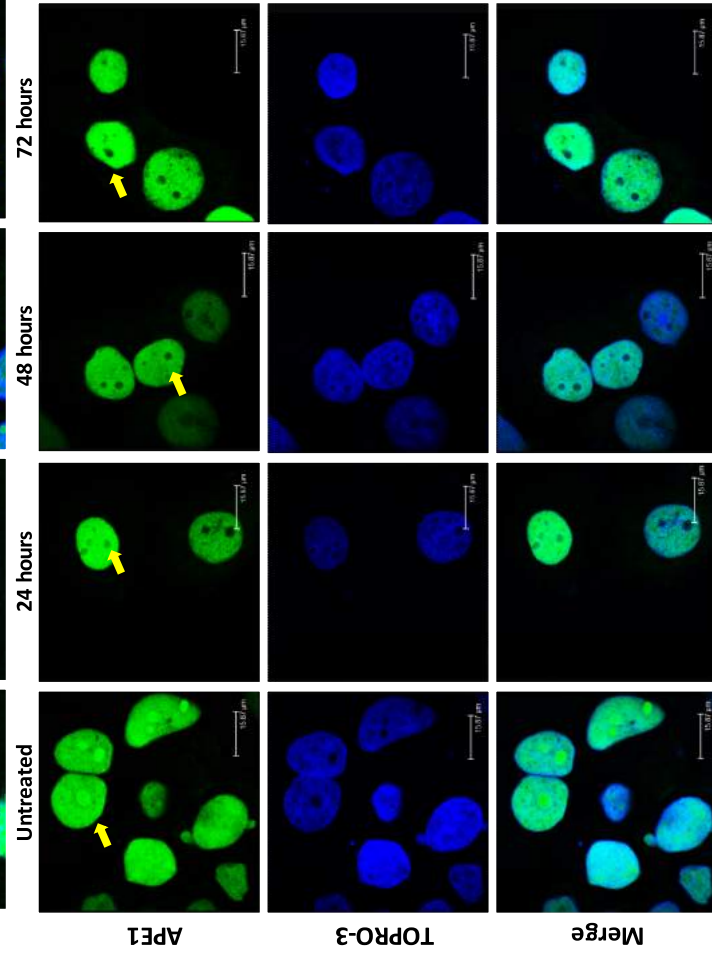

Supplementary Figure S1

C

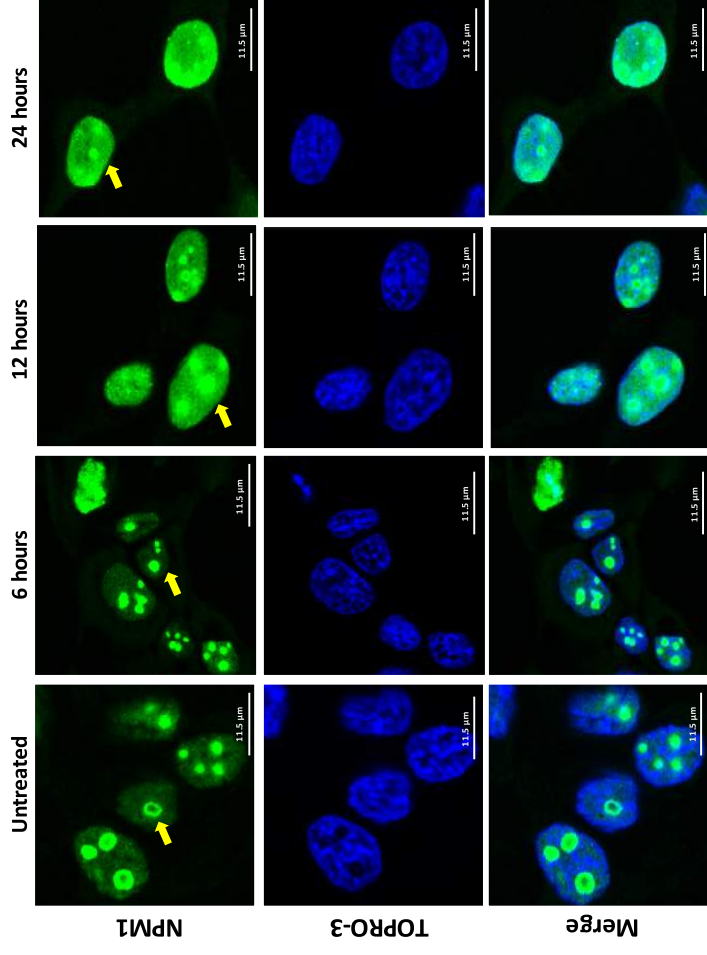

D

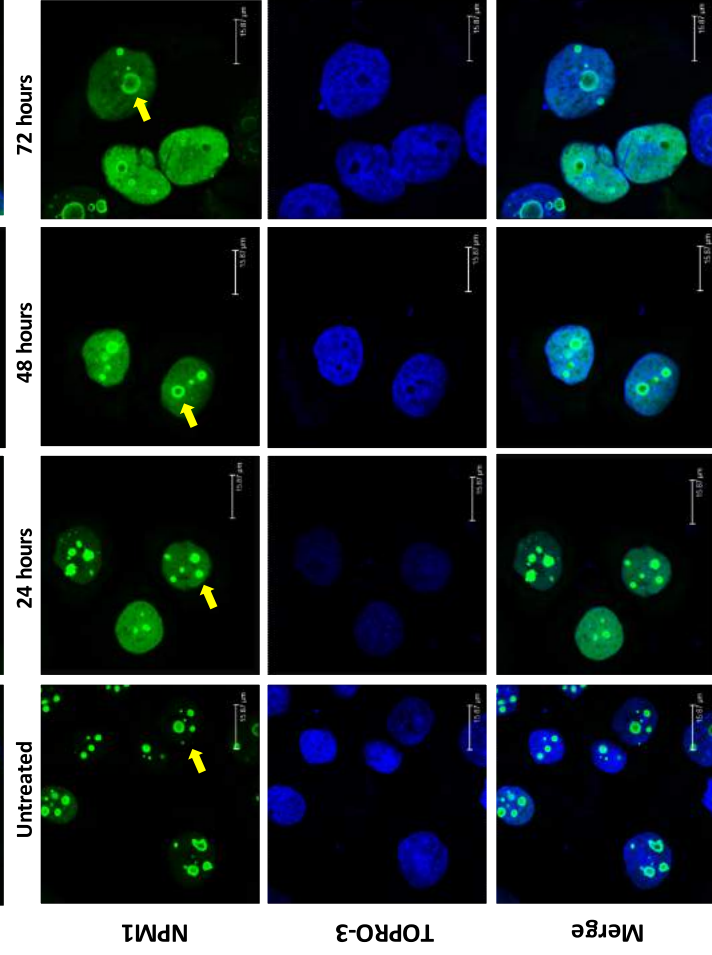

Supplement: Supplementary file 2 — Figure S1. CDDP induces a nucleolar emptying of APE1 and a nucleoplasmic re - localization of NPM1. (a, b) Immunofluorescence staining for APE1 on HCC70 (a) and HCC1937 (b) cells shows an evident nucleolar emptying of APE1 upon CDDP treatment (3.125 μM) in a time-dependent trend. The nucleolar repopulation is observed in HCC70 cells only, after 24 h of treatment. HCC1937 cells responded differently, with a prolonged emptying of nucleolar APE1 that persisted up to 72h of treatment. (c, d)Immunofluorescence staining for NPM1 on HCC70 (c)and HCC1937 (d) cells shows an increased accumulation of NPM1 into the nucleoplasmic compartment upon CDDP treatment (3.125 μM) in a time-dependent trend. Yellow arrowheads highlight representative cells showing the characteristic phenotype as described. ‘Untreated’ corresponds to the condition in which the cells didn’t undergo any treatment at the corresponding longer point of time. TOPRO-3 staining was used to identify nuclei. ‘Merge’ indicates the overlapping of the signals of APE1 (or NPM1) and TOPRO-3. (PDF 968 kb) [file 13046_2019_1294_MOESM2_ESM.pdf]

Supplementary Figure S2

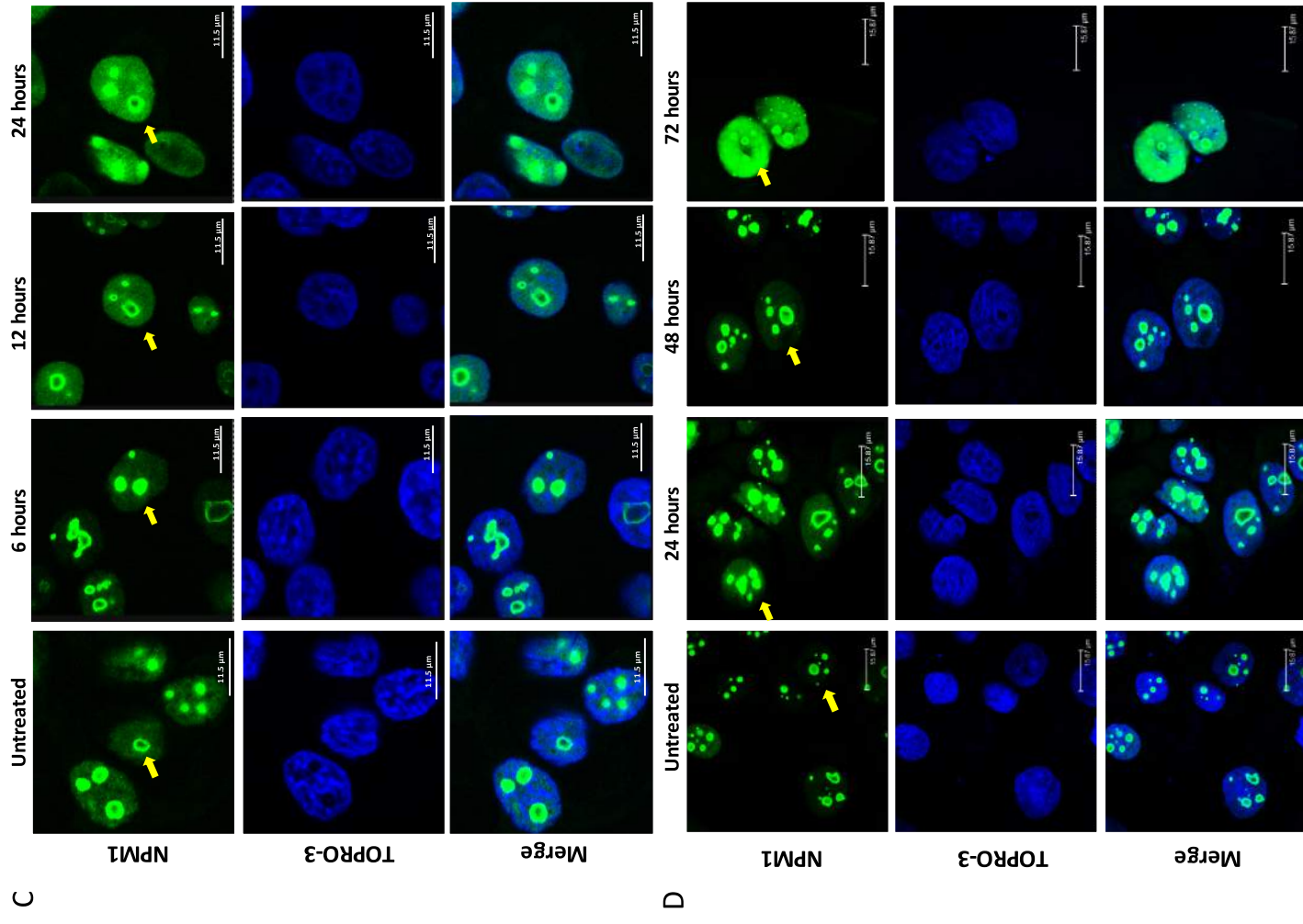

Supplementary Figure S2

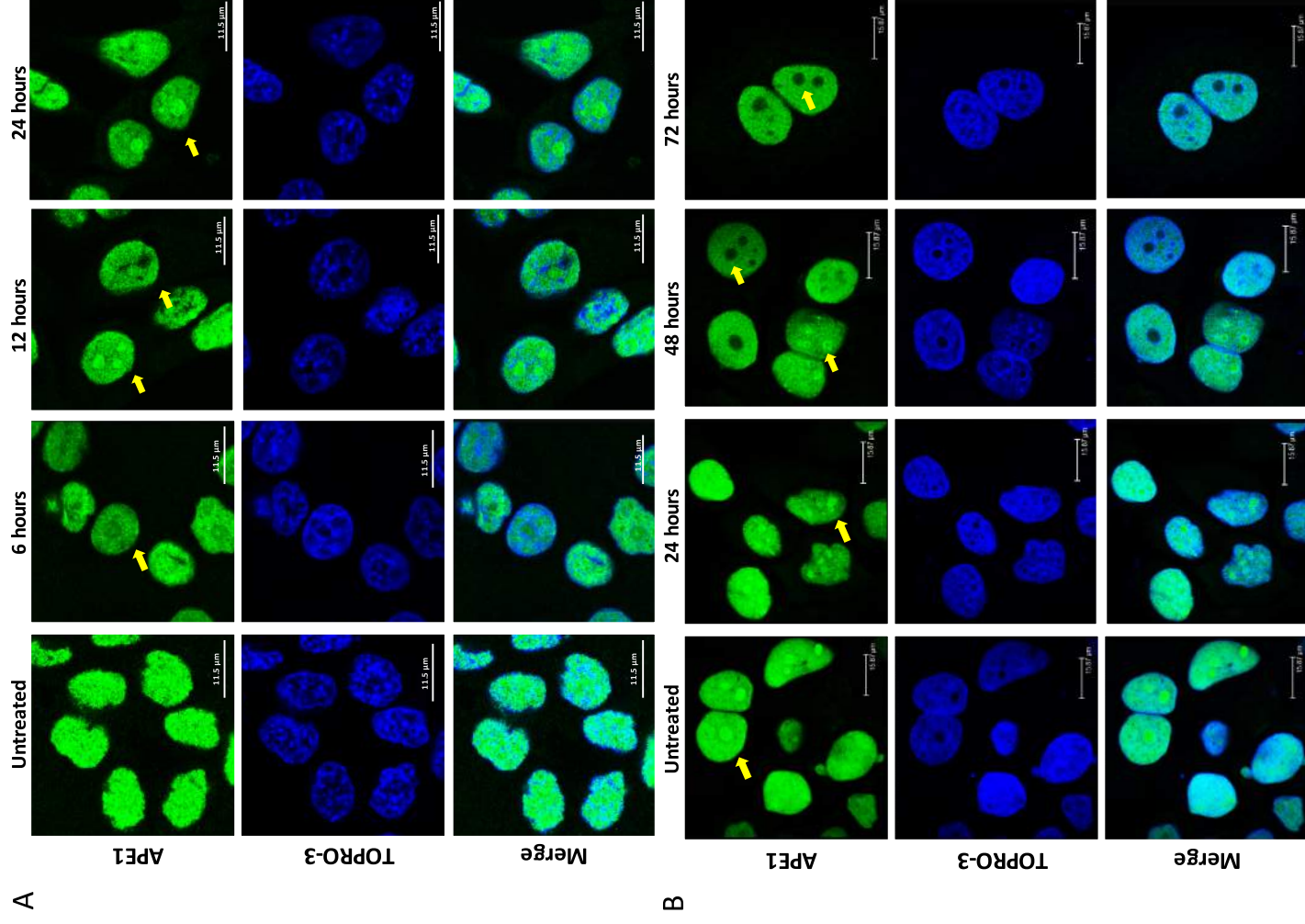

Supplement: Supplementary file 3 — Figure S2. CBDCA induces a nucleolar emptying of APE1 and a nucleoplasmic re - localization of NPM1. (a, b)Immunofluorescence staining for APE1 on HCC70 upon CBDCA treatment (100 μM)(a) and HCC1937 upon CBDCA treatment (25 μM) (b)cells shows an evident nucleolar emptying of APE1 in a time-dependent trend. The nucleolar repopulation is observed in HCC70 cells only, after 24 h of treatment. HCC1937 cells responded differently, with a prolonged emptying of nucleolar APE1 that persisted up to 72h of treatment. (c, d) Immunofluorescence staining for NPM1 on HCC70 upon CBDCA treatment (100 μM) (c)and HCC1937 upon CBDCA treatment (25 μM) (d) cells shows an increased accumulation of NPM1 into the nucleoplasmic compartment in a time-dependent trend. Yellow arrowheads highlight representative cells showing the characteristic phenotype as described. ‘Untreated’ corresponds to the condition in which the cells didn’t undergo any treatment at the corresponding longer point of time. TOPRO-3 staining was used to identify nuclei. ‘Merge’ indicates the overlapping of the signals of APE1 (or NPM1) and TOPRO-3. (PDF 999 kb) [file 13046_2019_1294_MOESM3_ESM.pdf]

Supplementary Figure S3

A

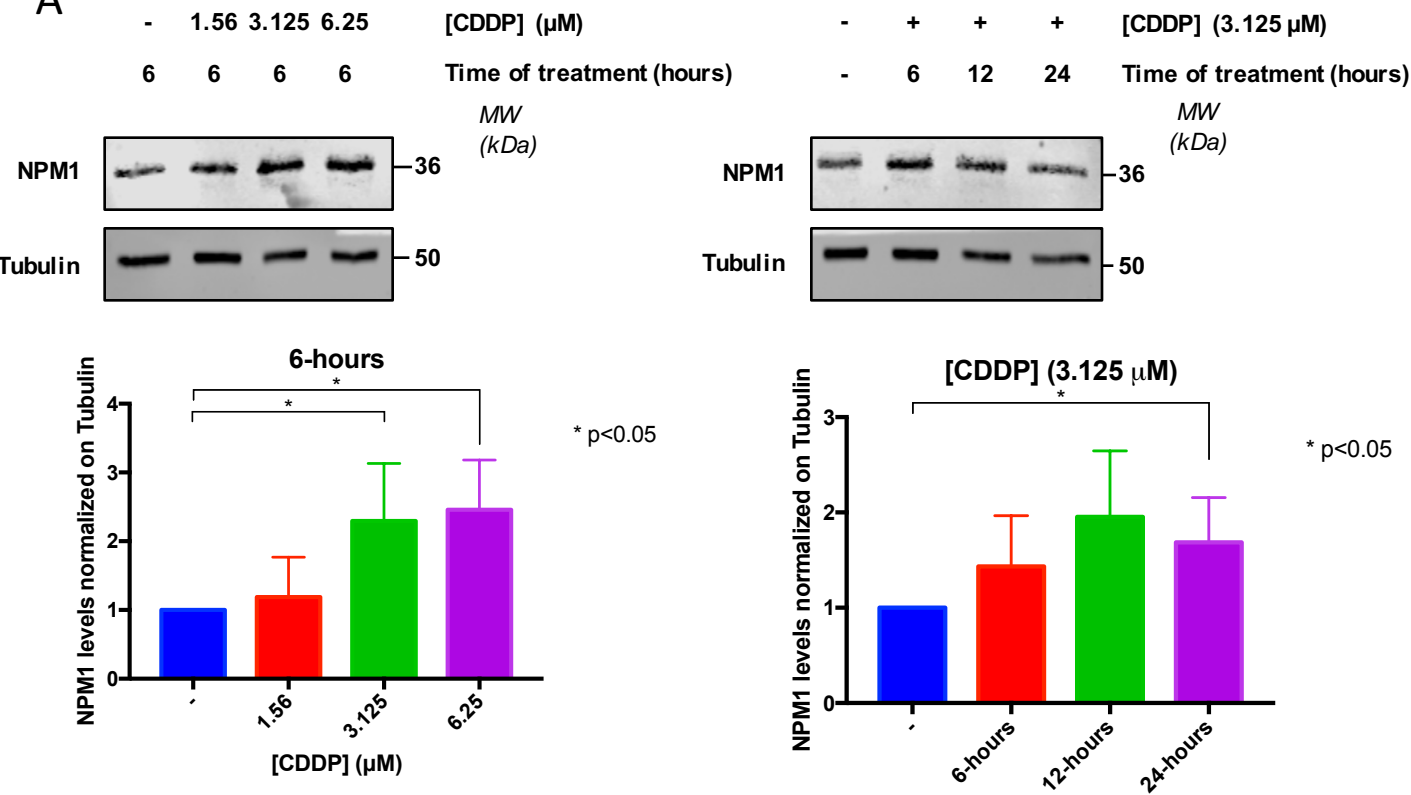

B

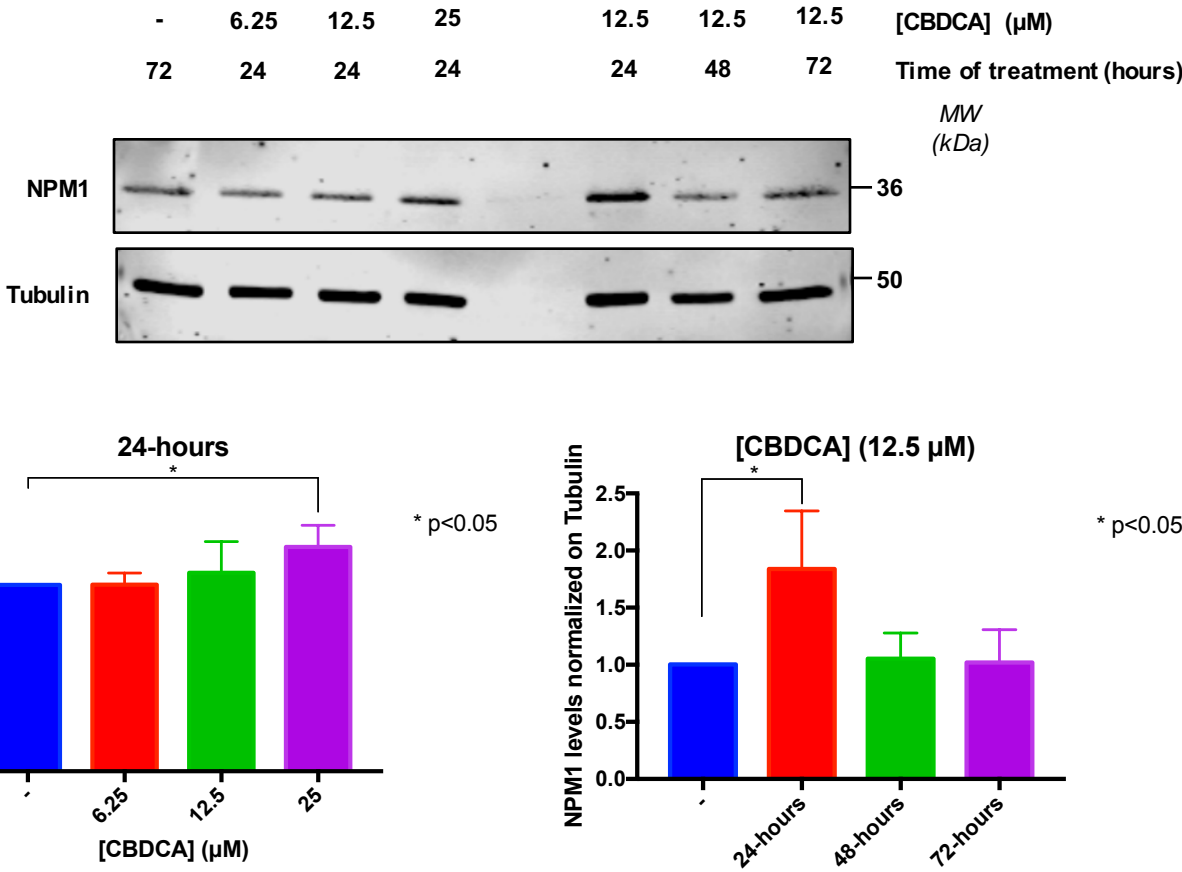

Supplement: Supplementary file 4 — Figure S3. Chronic treatment with Pt-compounds induces an increase of NPM1 on HCC70 cells. (a) (up) Representative western blotting shows the NPM1 protein levels trend in HCC70 cells treated with CDDP at different time points (right) or different concentrations (left), as specified on the top of the panel. On the right side of each panel, the Molecular Weights (MW), expressed in kDa, are indicated. On the left side of each panel, specific antibodies, used in the immunoblotting, are indicated. (bottom) Histograms reporting the quantitative values corresponding to the NPM1 protein amounts compared to the basal untreated conditions and normalized on Tubulin. Values express the mean viability ± SD from at least three independent replicates. *p < 0.05. (b) (up) Representative western blotting shows the NPM1 protein levels in HCC70 cells, treated with CBDCA at different concentrations or different time points, as specified on the top of the panel. On the right side of each panel, the Molecular Weights (MW), expressed in kDa, are indicated. On the left side of each panel, specific antibodies, used in the immunoblotting, are indicated. (bottom) Histograms reporting the quantitative values corresponding to the NPM1 protein amounts compared to the basal untreated conditions and normalized on Tubulin. Values express the mean viability ± SD from at least three independent replicates. *p < 0.05. (PDF 587 kb) [file 13046_2019_1294_MOESM4_ESM.pdf]

Supplementary Figure S4

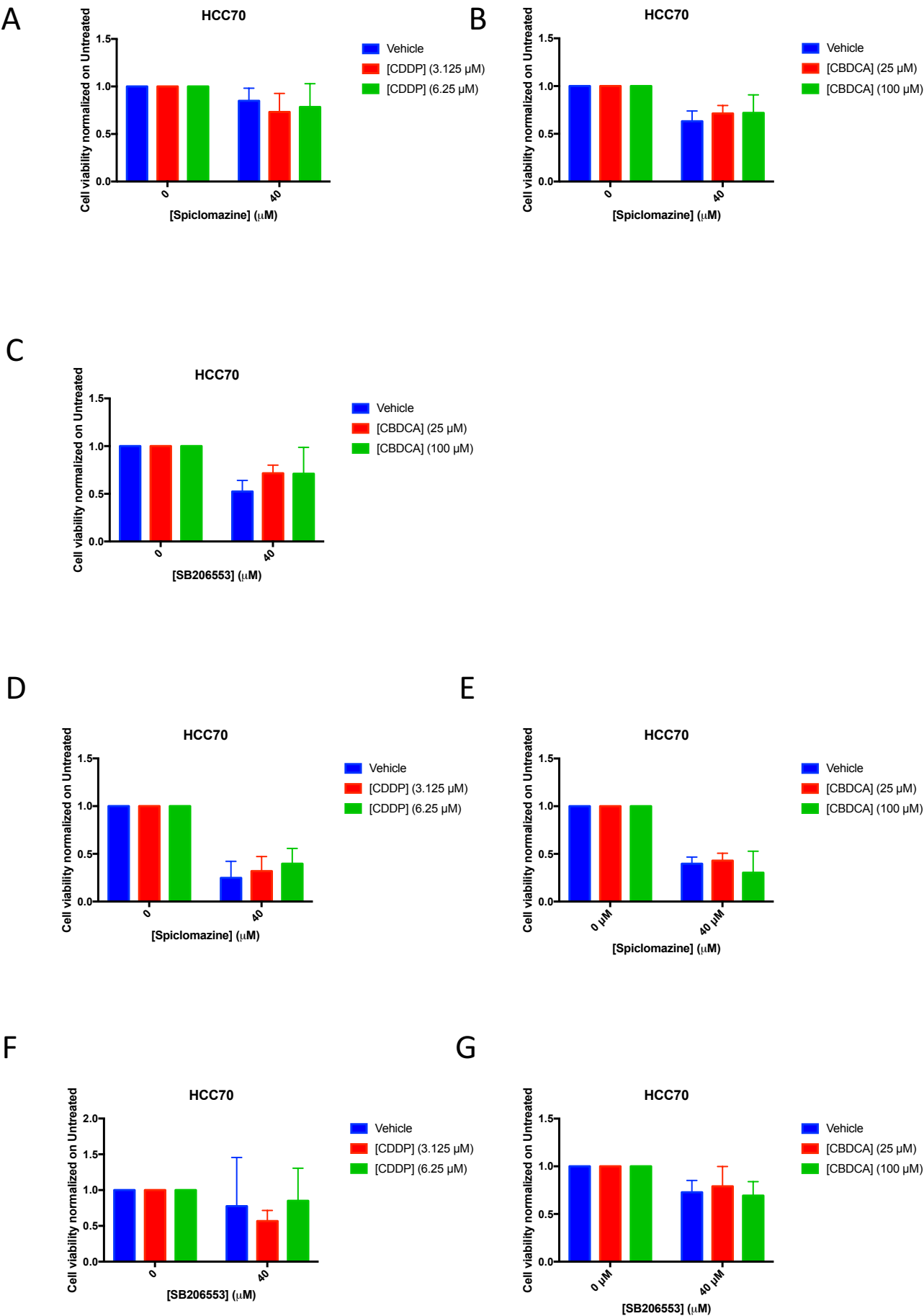

Supplement: Supplementary file 5 — Figure S4. Spiclomazine inhibitor does not sensitize HCC70 cells to Pt-compounds treatment. Histograms show the effect of co-treatments with CDDP (a, d, f) or CBDCA (b, c, e, g) and APE1/NPM1 inhibitors, Spiclomazine (a, b, d, e) and SB206553 (c, f, g)on HCC70 cells viability. Values express the mean viability ± SD from at least three independent replicates. Each value is normalized to the untreated condition. (PDF 81 kb) [file 13046_2019_1294_MOESM5_ESM.pdf]

# Supplementary Figure S5

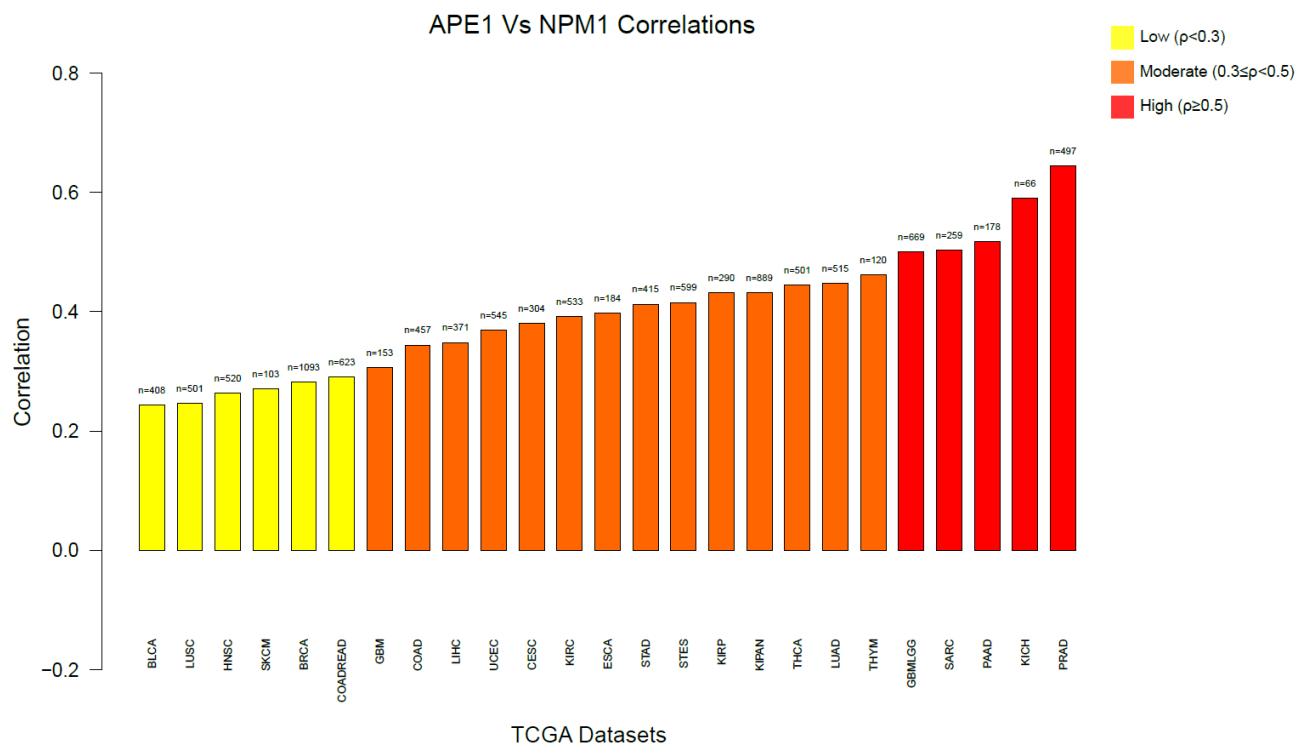

Supplement: Supplementary file 6 — Figure S5. Comparison of APE1 and NPM1 gene expression levels in TCGA tumor datasets. Barplot summarizing the statistically significant correlations (p ≤ 0.05) existing between APE1 and NPM1 expression levels in twenty-five TCGA tumor samples. RNA-seq data for thirty-six TCGA datasets were extracted and Pearson correlation coefficients between APE1 and NPM1 were calculated on a per sample basis, retaining and plotting only the statistically significant results. The number of patients profiled in every dataset is shown on top of each bar; bar colors reflect the size of the correlations. (PDF 96 kb) [file 13046_2019_1294_MOESM6_ESM.pdf]

Supplementary Figure S6

A

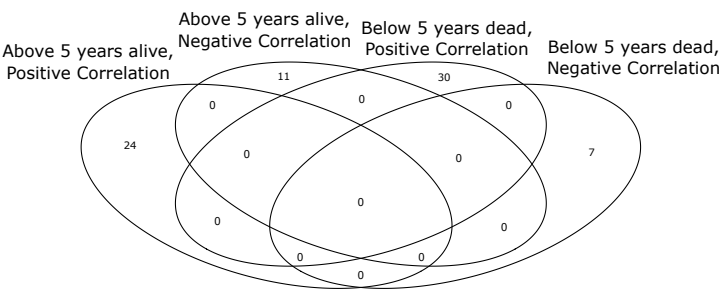

B

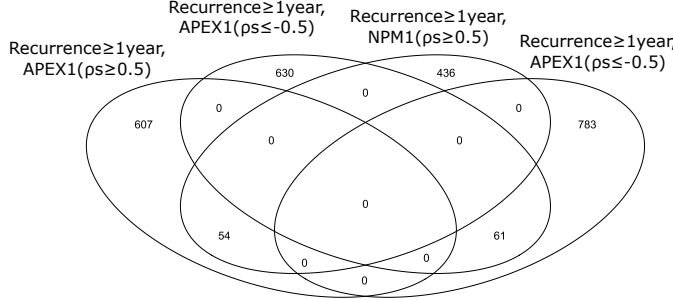

Supplement: Supplementary file 7 — Figure S6. Venn diagrams showing similarities and differences existing among the examined gene lists. Differences in patients’ overall survival below or above 5 years allowed to identify four gene signatures that positively or negatively correlated with both APE1 and NPM1 gene expression (a). Differences in disease recurrence after at least one year from diagnosis defined several specific signatures as well as two lists of genes that had a positive or negative correlation with both APE1 and NPM1 gene expression (b). (PDF 105 kb) [file 13046_2019_1294_MOESM7_ESM.pdf]
